# Digital research data: from analysis of existing standards to a scientific foundation for a modular metadata schema in nanosafety

**DOI:** 10.1186/s12989-021-00442-x

**Published:** 2022-01-05

**Authors:** Linda Elberskirch, Kunigunde Binder, Norbert Riefler, Adriana Sofranko, Julia Liebing, Christian Bonatto Minella, Lutz Mädler, Matthias Razum, Christoph van Thriel, Klaus Unfried, Roel P. F. Schins, Annette Kraegeloh

**Affiliations:** 1 INM - Leibniz Institute for New Materials, Campus D2 2, 66123 Saarbrücken, Germany; 2 FIZ Karlsruhe – Leibniz Institute for Information Infrastructure, Hermann-von-Helmholtz-Platz 1, 76133 Eggenstein-Leopoldshafen, Germany; 3 IWT - Leibniz-Institut für Werkstofforientierte Technologien, Badgasteiner Str. 3, 28359 Bremen, Germany; 4 IUF - Leibniz Research Institute for Environmental Medicine, Auf’m Hennekamp 50, 40225 Düsseldorf, Germany; 5 IfADo - Leibniz Research Centre for Working Environment and Human Factors, Ardeystraße 67, 44139 Dortmund, Germany

**Keywords:** Research data management, Toxicology, Nanomaterial, Minimum information standard, Metadata, Nanoparticle, Data re-use, Quality criteria

## Abstract

**Background:**

Assessing the safety of engineered nanomaterials (ENMs) is an interdisciplinary and complex process producing huge amounts of information and data. To make such data and metadata reusable for researchers, manufacturers, and regulatory authorities, there is an urgent need to record and provide this information in a structured, harmonized, and digitized way.

**Results:**

This study aimed to identify appropriate description standards and quality criteria for the special use in nanosafety. There are many existing standards and guidelines designed for collecting data and metadata, ranging from regulatory guidelines to specific databases. Most of them are incomplete or not specifically designed for ENM research. However, by merging the content of several existing standards and guidelines, a basic catalogue of descriptive information and quality criteria was generated. In an iterative process, our interdisciplinary team identified deficits and added missing information into a comprehensive schema. Subsequently, this overview was externally evaluated by a panel of experts during a workshop. This whole process resulted in a minimum information table (MIT), specifying necessary minimum information to be provided along with experimental results on effects of ENMs in the biological context in a flexible and modular manner. The MIT is divided into six modules: general information, material information, biological model information, exposure information, endpoint read out information and analysis and statistics. These modules are further partitioned into module subdivisions serving to include more detailed information. A comparison with existing ontologies, which also aim to electronically collect data and metadata on nanosafety studies, showed that the newly developed MIT exhibits a higher level of detail compared to those existing schemas, making it more usable to prevent gaps in the communication of information.

**Conclusion:**

Implementing the requirements of the MIT into *e.g.,* electronic lab notebooks (ELNs) would make the collection of all necessary data and metadata a daily routine and thereby would improve the reproducibility and reusability of experiments. Furthermore, this approach is particularly beneficial regarding the rapidly expanding developments and applications of novel non-animal alternative testing methods.

**Supplementary Information:**

The online version contains supplementary material available at 10.1186/s12989-021-00442-x.

## Background

Nanosafety research generates large amounts of data on various engineered nanomaterials (ENMs) from numerous studies. For an efficient nanosafety assessment, such data need to be preserved, disseminated, and made available for re-use by others [1]. To provide data-driven solutions in the field of nanosafety, data digitization needs further development. Nanosafety assessment involves the integration of data on materials properties along the life-cycle, hazard assessment, including mechanisms of action and dose–response relationships, as well as information on external and internal exposure. Such data contain descriptive information about ENM characterization and further specialized methods, ENM dosage and exposure routes, biological models, in vitro or in vivo test systems, and the readout of these assays. Thus, due to its multidisciplinarity, nanosafety research involves generation of complex and large data sets. In addition, there are different data requirements related to regulatory or scientific objectives and scopes. Currently, nanosafety research poses the challenge that many questions cannot be answered using existing and established test guidelines. This implies that methods have to be checked for their applicability and, if necessary, adapted [2–4]. Furthermore, due to the ongoing transition in toxicology from in vivo testing to the use of in vitro assays [5, 6], there is a demand for the development of advanced test systems in accordance with the 3R principle for replacement, reduction, and refinement of animal testing [7]. Alternative testing strategies can help to justify the study design and identify the impact of ENMs on specific endpoints [8, 9]. For example, the Adverse Outcome Pathway (AOP) concept links molecular initiating events to adverse outcomes mediated by a series of key events and therefore provides information regarding a toxicological response. AOPs are increasingly considered and used in regulatory settings for hazard and risk assessments of ENMs [10–13]. There are also proposals for concern-driven approaches to identify specific information needs for a given ENM based on the exposure scenario [14]. These needs could be addressed by a tiered structure, comprising primarily basic physicochemical investigations and evaluation of existing data and, secondly, the performance of a limited set of in vitro and in vivo studies for possible read across. Efficient use of such data could help to identify information gaps and the need for further studies [14].

The paradigm shift towards advanced in vitro test systems enables the use of high throughput screening (HTS) for the analysis of multiple assay endpoints related to genes, pathways or cell functions and is applicable for various model systems [15]. HTS results in the generation of large data sets which require supporting data processing techniques to identify significant assay read-out for ENM toxicity assessment [16]. Especially in the field of toxicological risk assessment, the use of “-omics” data and their systematic integration into a growing number of AOPs will further reinforce the need for advanced and integrated data management tools [17]. Therefore, in silico methods like computational modelling and machine learning are developing key components to support data analysis [18]. The required and resulting data can assist read-across approaches and grouping of ENMs based on information about ENMs physicochemical properties, in vivo and in vitro biological assays, or toxicological data (REACH No 1907/2006). Such computer-based information contributes to the prioritization and selection of necessary studies for the safety assessment of ENMs, offering new opportunities in view of limited testing resources and ethical aspects in animal testing (REACH No 1907/2006, OECD ENV/JM/MONO(2007)2). To keep up with the growing demands on ENMs and their safe use, digitization and FAIRification of research data is essential. FAIRification addresses the implementation of the FAIR Guiding Principles that are designed to ensure the Findability, Accessibility, Interoperability and Reusability of research data [19]. An important aspect of realizing FAIRification of data is the use of globally unique and persistent identifiers (PIDs) that enable long-lasting and reliable referencing of digital resources and thus ensure their findability by humans and machines. In general, a PID is a unique identifier (consisting of alphanumeric characters) provided by a service that guarantees that the identifier will still be resolved correctly, even if the location changes. Prominent examples of identifier systems are DOI (https://www.doi.org/) for identifying published scientific literature or associated data, or ORCID (https://orcid.org/) for identifying persons. Currently, in the field of nanosafety, data is mostly used for regulatory purposes or scientific publications with very limited or even restricted data accessibility. Much of the generated meta(data) is not published, remaining in laboratory notebooks (mostly analogically) or institutional or private electronic storage devices. This causes a gap between data and the availability of descriptive information. Data within scientific publications mostly includes tables and figures, which are not machine-readable and can be barely used to derive or generate an electronic data set. There is an increasing need for transparency and open accessibility to research results to improve the reproducibility and reusability of nanosafety research findings. Although scientific journals increasingly ask for provision of original research data and support provision of detailed additional information on experimental procedures and data analysis, most published articles provide only processed data presented as figures and tables, limiting findability, accessibility, and reuse. Regarding the storage of original meta(data), suggestions on potential comprehensive databases/repositories are still rarely provided to authors. However, providing data across disciplines is regarded as a prerequisite for multidisciplinary data sharing and improved data re-use [20]. Successful integration of (meta)data into databases as well as their re-use require detailed information and data completeness [21] paired with careful data curation. In the field of nanosafety, only a limited number of databases/repositories exist and most of them are not publicly accessible. Among them, eNanoMapper provides data about ENM characterization and biological and toxicological investigations. So far, eNanoMapper is regarded as the most FAIR-compliant database in nanosafety [22]. Recently, Comandella et al. [23] analyzed the availability and completeness of existing data and metadata on physicochemical properties of 18 ENMs from the eNanoMapper database. Data completeness was assessed by information checklists from the EU-funded projects NANoREG and GRACIOUS. The analysis revealed significant gaps in the availability of metadata, in particular related to ENM characterization and lacking application of standard operating procedures (SOPs) or more generally due to non-harmonized reporting standards. It can be expected that such gaps are also existent in metadata sets on toxicological assessment [23].

Metadata standards are needed to ensure consistency and effectiveness of databases. Metadata standards or ontologies are developed to support data annotation in specific databases, *e.g.*, eNanoMapper Ontology (eNanoMapper database), or derived from already established metadata standards *e.g*., Dublin Core (NanoHub) or ISA-Tab (ToxBank). The general use of databases in the field is currently limited due to differences in data structure and terminology, restricted upload possibilities and lack of sustainable financial support. Furthermore, metadata standards can be used to support digital data and metadata capturing and the organization of digital data and metadata along the whole data lifecycle spanning from the design of a study and performance of experiments to publication and storage in a repository. For example, at points of data transfer or at “curation boundaries” (see Table S3 for definition of the term), curation criteria are needed to evaluate the data quality and their description with metadata in order to facilitate reuse of such data. Specifically, the use of data in the regulatory context, requires high quality and reliability.

Complete, high quality, and re-usable data sets need to be based on standards or guidelines, specifying the information that needs to be reported [18]. To advance digitization in the area of nanosafety, modular description standards for multidisciplinary use are urgently needed. In this publication, existing standards, and guidelines, comprising regulatory guidelines (*e.g*.*,* OECD), guidelines for specific experiments (*e.g.,* ARRIVE [24]), and scientific guidelines (*e.g.,* MIRIBEL [25]) have been analyzed to create a catalog of descriptive information and quality criteria. However, the identified information should not be seen as a conclusive collection, as it would not be feasible to list all available standards or guidelines. The catalogue was further structured into six main modules with module subdivisions resulting in a minimum information table (MIT). A mapping analysis was performed in order to examine the extent of coverage of the MIT by existing open standards and ontologies (*e.g.,* eNanoMapper and ISA-Tab-Nano). The resulting concept is a modular and flexible data schema for data collection and curation. Further implementation steps are indicated and exemplified by identifying and integrating parameters with specific importance for advanced in vitro models of the intestine and the skin.

## Main text

### Sources for a minimum information schema

The use of standards in nanosafety research is crucial to ensure the relevance, reliability, reusability, and comprehensibility of datasets. Regulatory standards or guidelines comprise documents from official authorities that are designed to identify and characterize potential risks posed by ENMs with the aim of obtaining their regulatory approval or their notification. Regulatory standards find their main area of application in the field of ENM industrial development and are used for assessing and evaluating the safety of a product. However, nanosafety research further requires mechanistic understanding and predictive capabilities, in order to advance concepts such as AOP, grouping and read across, which cannot be addressed by existing regulatory standards or guidelines. The ongoing development of new test strategies and advanced test systems is a challenge for scientific research in the field (see “Results of the compatibility analyses” section). Alternative testing in accordance with the 3R principle leads to the development of a growing number of in vitro test methods. The development, standardization, and validation of these innovative methods cannot be achieved in accordance with existing regulatory standards or guidelines due to the limited scope of the latter. Therefore, scientific standards or guidelines are under development and validated regarding regulatory acceptance [26]. Scientific standards are more closely oriented to scientific practice, aiming at either enhancing comparability and reproducibility and therefore the communication and exchange of results from ENM studies or describing specific requirements for certain methods (method standards). There are several organizations active in the development and definition of standards or guidelines for nanosafety assessment or related research fields (Table 1). Relevant information and recommendations should be given on:

- properties of ENMs regarding their application potential and their properties under biologically/physiologically relevant conditions
- biological modes of action and toxicologically relevant impact of ENMs
- detection of ENMs in biological systems to determine initiating events and internal exposure at the target site
- requirements on data for hazard and exposure assessment
- validation of in vitro assays by in vivo experiments
- development of regulatory relevant innovative test systems and predictive assays

**Table 1:** Existing standards and guidelines

Organization/project name	Objectives of the organization/project	Name of specific standard/guideline used in this project	Content of the standard/guideline
Organization for Economic Cooperation and Development (OECD) Working party on Manufactured Nanomaterials (WPNM)	OECD WPNM working on the development of methods and strategies to identify and manage the potential health and environmental risks of nanomaterials. Review the current OECD test guidelines for applicability to ENMs. Identify the need to develop new test guidelines	Testing Program of Manufactured ENMs Dossier on Titanium Dioxide—PART 1/1; 1/2; 1/3 [80]	Document presents the Dossier of the Titanium Dioxide (TiO2) manufactured ENMs
		OECD GD 211 [81]	Guidance Document for Describing Non-Guideline In Vitro Test Methods
International Organization for Standardization (ISO) Technical Committee (TC) 229	Special issues on nanotechnologies	ISO/TC 229 (free available preview):	This Technical Report provides guidance for the physicochemical characterization of manufactured nano-objects and their aggregates and agglomerates (NOAA) greater than 100 nm presented for toxicological testing to aid in assessing and interpreting the toxicological impact of manufactured nano-objects and to allow the material under test to be differentiated from seemingly similar materials
		ISO/TR 13,014:2012(en) Nanotechnologies — Guidance on physico-chemical characterization of engineered nanoscale materials for toxicologic assessment [47]	
EU’s regulation committee: Registration, Evaluation, Authorization and Restriction of Chemicals (REACH)	Registration of substances that are manufactured or imported at a rate higher than one ton per year. These substances include ENMs with a focus on industrial scale production	REACH regulation on nanoforms [48]	Lists a range of physicochemical parameters necessary for the registration of ENMs. Including intrinsic and system-dependent material properties
Minimum Information Reporting in Bio–Nano Experimental Literature (MIRIBEL)	MIRIBEL describes the minimum information, including a reporting checklist which should contribute strongly to reproducibility, quantitative research, and communication of scientific results	Scientific publication [25]	Lists three categories to be reported: materials characterization, biological characterization, and details of experimental protocols
DaNa	DaNa project developed a methodology for selecting, recording, and evaluating existing toxicological publications	DaNa Checklist [82]	A checklist provides a collection of criteria which should be included: physicochemical characteristics of ENM, sample preparation, test-parameter and general information on data analysis and statistics (checklist)
In vitro approaches to assess the hazard of ENMs	Review article: Broadly discuss the current state-of the art ENM hazard assessment strategies using in vitro approaches	Scientific publication [3]	The main topics are the preparation and physicochemical characterization of ENM, choice and characterization of the cellular models, simulation of a realistic exposure and dosimetry, use of controls, and the cellular response readout. This information provides important recommendations for the planning, implementation, and analysis of nanosafety studies
National Centre for the Replacement, Refinement and Reduction of Animals in Research (NC3Rs)	Replacement, Refinement and Reduction of Animals in Research	Animal Research: Reporting of In Vivo Experiments (ARRIVE); The ARRIVE guidelines 2.0: author checklist [34]	Checklist of recommendations to improve the reporting of research involving animals – maximising the quality and reliability of published research, and enabling others to better scrutinise, evaluate and reproduce it
Library of Integrated Network-Based Cellular Signatures (LINCS)	Supplies description standards with a focus on reagents, assays, and experiments	Metadata Specifications: Cell Lines [83]	Metadata specifications for assays and experiments
Template for the description of cell-based toxicological test methods to allow evaluation and regulatory use of the data	Detailed toxicity test method template (ToxTemp)	Scientific publication [84]	Template for the Description of Cell-Based Toxicological Test Methods to Allow Evaluation and Regulatory Use of the Data
Minimum Information about a Flow Cytometry Experiment (MIFlowCyt)	Supports storage, annotation, analysis, and sharing of flow cytometry datasets	Scientific publication [58]	Minimum Information about flow cytometry experiments
Reproducibility in light microscopy: Maintenance, standards and SOPs	Recommendations for quantitative microscopy data and reproducible results	Scientific publication [56]	Method specific guidance on the use of light microscopy
4D Nucleome Initiative (4DN) Imaging Standards Working Group (IWG)	Working in conjunction with the BioImaging North America (BINA) Quality Control and Data Management Working Group (QC-DMWG), propose light Microscopy Metadata specifications that scale with experimental intent and with the complexity of the instrumentation and analytical requirements	Scientific publication [57]	Minimum information for fluorescence microscopy—The proposal is an extension of the OME data model and aims at increasing data fidelity, ease future analysis, and facilitate objective comparison of different datasets, experimental setups, and essays

Summary of organizations and projects active in the development of standards or guidelines for nanosafety and related research fields. These standards or guidelines have been used in this study for the analysis of relevant descriptive information

Existing standards and guidelines (see below) were analyzed according to this content.

The Organization for Economic Cooperation and Development (OECD) Working Party on Manufactured Nanomaterials (WPNM) is leading an international collaboration, working on the development of methods and strategies to identify and manage the potential health and environmental risks of ENMs [27]. A large set of OECD test guidelines is available, and major advances were made regarding the evaluation of their applicability to ENMs testing. Additionally, several new test guidelines, protocols for sample preparation, strategies for risk assessment and elaboration of basics for exposure assessment were and are being developed [28]. The applicability and validity of the test guidelines were examined within a sponsorship program for the testing of ENMs. While these test guidelines mostly provide in-depth information and recommendations regarding study design and test model acceptance criteria that are applicable or adaptable to ENMs, there are still gaps that need to be identified and filled (*i.e.* physicochemical characterization, hazard, fate and risk assessment) to enhance the applicability of methods and data to nanosafety research [3].

In this context, quality criteria are characteristics that are typically expected to be fulfilled by the test system and necessary for the evaluation and curation of corresponding datasets. For example, the use of appropriate positive and negative controls is one prerequisite for the interpretation of an assay and a criterion for the functionality of an assay [3]. To obtain meaningful results from in vitro and in vivo studies, the test conditions must be in context with relevant human exposure scenarios. The development of ENMs is rapidly increasing due to their use in various fields including technical fields, medicine, food, and cosmetics. ENMs released during their life-cycle may affect the environment and human health, depending on the nature and quantity of the ENM [29]. Following the AOP concept, there is an obvious need to determine the distribution and fate of ENMs inside the human body [30–33] as this information is relevant for the identification of ENM induced adverse effects as a result of cellular and molecular mechanisms of action [10]. Therefore, adverse effects induced by ENMs should be considered in the context of realistic human exposure [34] and moreover the dose of ENM delivered to the target site [35]. However, data on actual exposure and dosimetry are unavailable for the whole range of different ENMs [36]. In line with the AOP concept, dosimetry needs to consider the distribution of ENMs at the various levels from the various organs down to tissue, single cell, and even subcellular level. Accordingly, models are available for the prediction of doses under various experimental conditions [37, 38]. Various approaches have been taken to quantify cell-associated or even intracellular ENMs [39–41]. However, to avoid misinterpretation of such results, an exact knowledge of the experimental parameters is necessary [42]. At the tissue level, information on clearance, biodissolution or biotransformation are relevant for mechanistic understanding and prediction of local ENM doses and retention [43].

Overall, standards are needed that provide information on the model system, test method, biological characterization, acceptance criteria of the test model, administered dose, study design and analysis methods. These should consider the three principal routes of (unintentional) exposure to ENM, *i.e.,* the dermal- (*e.g.,* in relation to ENMs in cosmetic products), the oral- (*e.g.,* related to ENMs in food products) and especially the inhalation-route [3].

OECD test guidelines are typically applied for regulatory purposes, *e.g.,* OECD TG 412 (Subacute Inhalation Toxicity). This test guideline is designed to fully characterize test chemical toxicity by inhalation and to provide robust data for quantitative risk assessment including a no-observed-adverse-effect concentration (NOAEC). It is widely used in regulatory and scientific studies of ENM ([44]; Series on the Safety of Manufactured Nanomaterials e.g., [45]). The guideline was adopted in 2017 to enable the testing of ENMs. The guideline describes the model system to be used for the investigation, as well as the type and implementation of the exposure. It specifies several parameters to be investigated and documented but it does not include any thorough description, *e.g.,* for the qualitative or quantitative analysis of ENM at the target site. As discussed above, information on exposure and dynamic doses at the target sites is necessary for the identification of ENM induced effects. Standards or guidelines defining requirements to evaluate ENM concentrations at target tissues and target cells would support these investigations but are not available. Considering the evaluation of mechanistic information, the OECD provides a “Users' Handbook Supplement to the Guidance Document for Developing and Assessing AOPs” which should be considered for the establishment of AOPs [46].

Another resource providing standards is the International Organization for Standardization (ISO) Technical Committee (TC) 229 with a focus on nanotechnologies. The ISO/TC 229 provide guidance on terminology and nomenclature, measurement and characterization, health, safety, and environment, and materials specifications for the study of ENMs [47]. Terminology and nomenclature define nanoobjects like nanoparticles, nanofibers, and nanoplates. The guidance for measurement and characterization focuses on procedures (*e.g.*, TEM, SEM, UV-­VIS-­NIR spectroscopy, and TGA) for the investigation of size, surface charge, and aggregation or agglomeration state. The guidance on health, safety, and environment focuses on toxicity testing methods, workplace health and safety measures and a variety of consumer products. The EU regulatory committee on Registration, Evaluation, Authorization, and restriction of Chemicals (REACH) is involved in the assessment and registration of substances that are manufactured or imported at a rate higher than one ton per year. These substances include ENMs with a focus on industrial-scale production. The updated REACH regulation on nanoforms [48] lists a range of physicochemical parameters necessary for the registration of ENMs. These include intrinsic and system-dependent materials properties. However, test methods to identify materials properties relevant for toxicological effects still need to be further defined to reach general acceptance. The regulatory standards or guidelines play an important role in the risk assessment and standardization in the field of nanosafety. However, none of these standard and guideline collections are complete in the sense that they deliver a complete set of parameters as well as specification of appropriate measurement approaches or methods.

An important topic in the scientific community of nanosafety is the improvement of methods standardization [49–51]. The scientific guideline MIRIBEL (Minimum Information Reporting in Bio–Nano Experimental Literature) lists three categories: materials characterization, biological characterization, and details of experimental protocols [25]. MIRIBEL describes the minimum information, including a checklist, which should contribute strongly to reproducibility, quantitative research, and communication of scientific results. MIRIBEL does not provide methodological protocols, *e.g.,* to detect ENMs at the target site. It represents a comprehensive approach for the description of ENM studies, in contrast to standards for individual methods. Furthermore, the DaNa project developed a methodology for selecting, recording, and evaluating existing toxicological publications. A checklist provides a collection of criteria which should be included: physicochemical characteristics of ENM, sample preparation, test-parameter and general information on data analysis and statistics (checklist). In addition, the DaNa project offers validated Standard Operating Procedures (SOPs), including the categories physicochemical properties, sample preparation, and biological test methods on its internet platform [52].

Overall, the DaNa knowledge base offers basic criteria for the quality assessment of published studies and a selection of potential methodologies for nanosafety. Drasler et al*.* published a review on "In vitro approaches to assess the hazard of nanomaterials" with comprehensive recommendations on the study design [3]. They are based on an analysis of methods in published studies to assess the biological response upon exposure to a diverse array of ENMs. Common and relevant biological in vitro methods and endpoints are listed and recommendations for their use are provided. The main topics are the preparation and physico-chemical characterization of ENM, choice and characterization of the cellular models, simulation of a realistic exposure and dosimetry, use of controls, and the cellular response readout [3]. This information provides important recommendations for the planning, implementation, and analysis.

Beside nanospecific guidelines, there are a variety of methods specific guidelines and standards. For the implementation and description of in vivo studies, the Animal Research: Reporting of In Vivo Experiments (ARRIVE) Guidelines are widely used. They are resulting from an interdisciplinary approach intended to improve the reporting of research using animals for maximizing published information while minimizing unnecessary studies [24]. The Library of Integrated Network-Based Cellular Signatures (LINCS) supplies description standards with a focus on reagents, assays, and experiments [53]. In contrast, method-specific guidelines provide important details and parameters that should be considered when applying the method. Microscopy examinations are a central element in nanosafety research. The basic characterization of ENMs *e.g.*, particle size, size distribution and morphology or the qualitative and quantitative identification of ENMs at the target site, including their fate and imaging of tissue responses requires the use of advanced imaging techniques [54, 55]. Method specific guidance on the use of light microscopy is given by Deagle et al*.* with a focus on reproducibility by highlighting the need of maintenance, standards, and SOPs for high quality microscopic data [56]. An approach to improve the quality of fluorescence microscopy is also recommended by the 4DN Imaging Standards Working Group [57] and has been published as “Minimum information guidelines for fluorescence microscopy”. The guidelines focus on required information to improve data consistency, simplification of follow-up studies and the comprehensibility of various study designs and results. Another example for method specific guidelines is the “Minimum information about a flow cytometry experiment (MIFlowCyt)” [58]. It supports storage, annotation, analysis, and sharing of flow cytometry datasets.

The quality of information not only contributes to regulatory processes but also increasingly influences the publication of research results. Therefore, journals like Particle and Fibre Toxicology, Chemical Research in Toxicology, ACS Nano, Archives of Toxicology, Nature Nanotechnology, and Nanotoxicology list information on data requirements in their author guidelines. However, the information content in journal guidelines is limited. In terms of good scientific practice, data quality and the use of quality criteria are fundamental for academic research to ensure high quality data for publications.

Overall, regulatory, and scientific standards or guidelines provide important requirements for the risk assessment of ENMs—although they are limited in their statements regarding the description of analytical procedures. In addition, specific properties of ENMs pose a challenge to many of the conventional methods, which are used for characterization. These limitations are critical aspects for the development of robust methods for the analysis of ENMs. All in all, the need for standardized reproducible methods which can be used for a wide range of ENMs and support their safety assessment is given.

### Derived minimum information table

The above-mentioned scientific and regulatory sources were analyzed, and the information used to create the basis for a collection of relevant parameters in a MIT. Additional parameters were added, according to the experience of the authors. The provided MIT (Additional file 2) was further completed after discussion with experts during a workshop organized in June 2020 [59]. As stated by Papadiamantis et al*.*, a universal schema capturing the complete metadata would need to be huge, highly complex, and seems unrealistic [18]. However, we consider this table as a necessary basis and starting point for nanosafety research and regulation. Depending on the specific research question or experimental setup, only a subset of parameters might be required, whereas additional parameters might be necessary, for example for the development or application of new approach methodologies (see Section “Results of the compatibility analyses” and Additional file 1: Table S2).

The parameters provide specific additional information and context to the data important to enhance the reproducibility and re-use of datasets. Therefore, they might also be designated metadata or subject-specific metadata. Starting from the structure provided in the MIT, a structured metadata schema could be developed. Metadata schemas represent a common consensus on the hierarchical and relational structure of the metadata, including rules regarding the naming of parameters and allowed content. They are used to make the related data portable, *i.e.,* independent of the recipient by introducing relations between all metadata. Well-known metadata schemas are for example designed for libraries (*e.g.,* Dublin Core Metadata Initiative, DataCite Metadata schema). In contrast, the CCLRC Scientific Metadata Model (CSMD) by Sufi and Matthews [60] is an example of a data-driven metadata model. The latter one aims at collecting information on the data generated at different levels (*e.g.,* programs, studies, investigations) in scientific studies, to allow such data to be re-used for parallel or follow-up studies. The CSMD supports indexing at different levels of granularity and the generic nature of the model allows it to be adapted to various scientific disciplines. In principle, the CSMD model could serve as the basis for a metadata schema to map the MIT. However, this requires an adaptation of the CSDM, especially regarding the subject-specific parameters that are currently not supported.

**Fig. 1:**
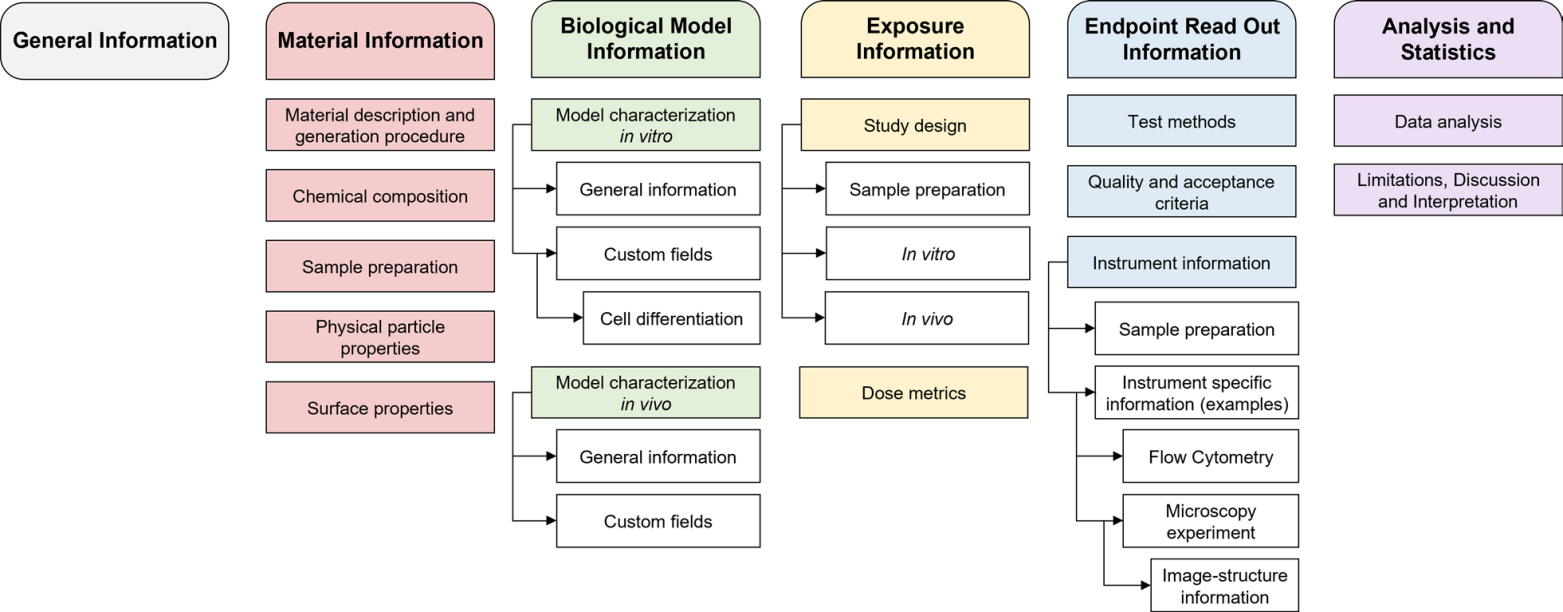
Overview of the structure of the MIT, divided into six main modules with module subdivisions. The comprehensive and detailed schema is available in the Additional file 2

With regard to an improved interoperability, the metadata schema may include different terms for the same circumstance. Therefore, the MIT lists synonyms *e.g.,* zeta potential and surface charge (see Supporting information, table). In general, metadata can be read by humans, however, some metadata are rather used for transient exchange between electronic systems [61]. The current schema keeps open the possibility to integrate machine generated metadata (*e.g.,* from experimental setups or measurement devices). The provided metadata are intended to be meaningful to researchers from different disciplines. The MIT is divided into six main modules with module subdivisions, as represented in Fig. 1. The modules are explained in the following sections.

#### General information

This module contains formal metadata, e.g., authorship or data generation date. In addition to subject-specific metadata, bibliographic information is normally used to describe digital resources like publications. This type of metadata is important for the description of data provenance or research on reference information. Among others, the Dublin Core Metadata Element Set Version 1.1, developed by the Dublin Core Metadata Initiative (DCMI) [62], or the DataCite Metadata Scheme from the DataCite International Consortium [63] are widely recognized for this purpose. If studies and corresponding data need to be stored in a database/repository, administrative and technical information are required together with metadata. Furthermore, some metadata in the DCMI and/or DataCite schema are not necessary for our purpose (*e.g.*, ‘Publisher’), which is less relevant than the data generating institution (*e.g.,* university, institute, company). Therefore, the suggested MIT aims to introduce relevant terms only.

#### Material information

The generation of ENMs for research purposes is based on empirical knowledge and usually follows clearly defined procedures to generate and investigate new materials. However, researchers mostly document their method in a way that differs from the notation used in Standard-Operating-Procedures (SOPs). Documentation is often performed analogically (pen and paper) and without an explicit formulation of implicit knowledge. Therefore, it is difficult to reproduce the experimental method. To assess safety aspects, the underlying material must be defined unequivocally to distinguish between variants. The same holds for the physical characterization of the material, which is not easy and often a complex issue. For instance, standard measurement methods like DLS (Dynamic Light Scattering) do not deliver information about particle shape and allow only statements with limited validity about size distribution. It is, therefore, important that all involved parties (not only the regulation agencies) get thorough information. Regarding precision, scope of application and some other quality measures of the characterization methods, we provide a summary of 40 characterization methods (Additional file 1: Table S1).

#### Biological model information

This group specifies the biological target (cells or organisms) exposed to the materials. The in vitro and in vivo model characterization requires detailed information, which is usually not or only partially provided in any publication. Such detailed information needs to be retrieved from other sources (*e.g.,* from online resources and/or the provider of the biological model), which is a cumbersome task. For researchers or regulatory authorities, this information is important as it influences the reproducibility of study results or re-use of data for risk assessment. Precise information on the cell line, its origin and genetic background is important, in addition to information on the cultivation details or differentiation status of the cells. Furthermore, detailed information about the in vitro cell culture system like plate format or the composition of the cell culture medium, including presence or absence of serum is important for the assessment and re-use of experimental results. Information about the used animal strain or the housing of experimental animals might even influence the decision-making of re-using data. Environmental enrichment of animal cages, for example, has a high influence on behavioral studies. Therefore, under this category, 69 parameters are requested regarding detailed information about used biological models and their handling.

#### Exposure information

According to the previous group, the requested parameters here are also divided into in vitro and in vivo related information, depending on the used biological model. In this group, specifications on how biological models are exposed to the ENM and to which amounts of ENM are requested, *e.g.,* details about the applied materials dose, route of exposure and type of application (e.g., as aerosol or dispersed), including sample preparation, delivered dose, or the study design in general. Implementing these MIT-parameters into an ELN as highly resolved SOPs could help to minimize the problem of unavailable implicit knowledge, which is often not reported and, thereby, improve data re-use and the reproducibility of experiments.

#### Endpoint read out information

This group specifies the test methods used to assess the material impact on the biological model and contains most of the metadata, mainly instrument specifications. These parameters are unique for the underlying method and vary considerably among different instruments. At the time of writing, three instruments are included (flow cytometry, light, and electron microscopy). Parameters of other methods can be added and will be incorporated in the metadata schema without redundancy after parameter unification. As mentioned above, all the fine-grained details should be implemented in the kind of highly resolved SOPs as measurement templates within the used ELN.

#### Analysis and statistics

The last group includes information about the data analysis methods and statistics, as well as the used software for data evaluation and known limitations of the study. If the analysis includes, for instance, concentration profiles from a high throughput screening assay, the result is typically a mathematically fitted curve describing the dose–response relationship, and all the underlying parameters to describe the fit must be given to understand what that result represents.

## Compatibility analysis and evaluation

**Fig. 2:**
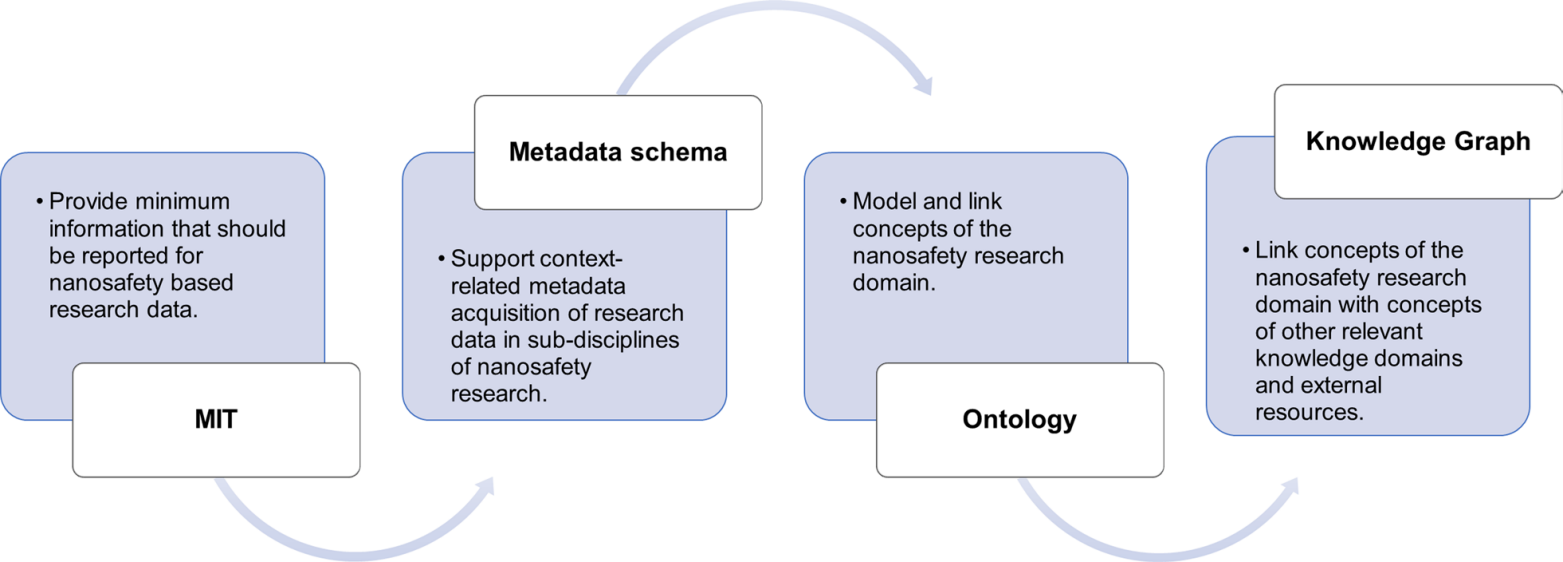
Description standards to be developed for nanosafety research data: The MIT defines minimal information to be reported. This information was used here to define the basis for a modular machine-readable metadata schema. The latter might be developed further into an ontology, representing the hierarchical structure and relationships between the various entities in a machine-readable format. Overarching interlinkage of ontologies from separate knowledge domains leads to the development of a knowledge graph

There are several options of mapping contextual metadata in a standardized manner, *e.g.,* by metadata schemas, controlled vocabularies, or ontologies (see Table S3 for definition of the terms). The derived MIT, which represents spreadsheet based minimum information, will be used as a starting point for the development of description standards for nanosafety research, including a machine-readable metadata schema (e.g., encoded in XML format), ontology, and knowledge graph (see Table S3 for definition of the terms) (Fig. 2). Thereby, a contribution can be made to increase the traceability, reliability, and re-use of enriched datasets.

To address the diversity of nanosafety research data, a module-based metadata schema seems to be appropriate. By such a modular schema, scientists in the field are supported in recording all metadata relevant for their intended use (this can be for example descriptive, subject-specific, and technical information, but also details on the formation context, the provenance, and the data quality) in a machine-readable manner throughout the entire research process. The metadata can then be stored together with the associated research data, which makes the research process transparent and comprehensible for re-users.

To explore whether the collected MIT (described in "Main text" section) could be mapped by existing open standards or ontologies, a literature survey was performed to define the state of the art. The survey was based on selective criteria such as the appropriateness for describing research data on nanosafety, the extensive compatibility with content of the MIT as well as the acceptance and dissemination within the community. As a result, two candidates were identified: the eNanoMapper ontology and the ISA-TAB-Nano standard (see detailed information in the Additional file 2). Subsequently, a mapping (see Table S3 for definition of the term) was carried out using the two selected test cases. The comparisons were made intellectually with the help of Excel sheets according to the Source-to-Target principle. The term “**labeled field**” (see Table S3 for definitions of the term) is used in the further course of the article as a synonym for the different terminologies used by the different standards.

## Results of the compatibility analyses

### One-to-one mapping

In the first step, a one-to-one mapping was carried out, including the names of all labeled fields defined in the MIT (labeled fields for grouping in total 33 e.g., General Information, content-bearing labeled fields in total 300 e.g., experiment name), to identify identical naming of the labels. An overlap of 34% with the eNanoMapper ontology (113 of 333 labeled fields of the MIT were identified in the eNanoMapper ontology) and 17% with the ISA-TAB-Nano specification (55 of 333 labeled fields of the MIT were identified in the ISA-TAB specification) was found, respectively. Hence, according to this first comparison the coverage between the MIT and eNanoMapper and ISA-TAB-Nano turned out to be rather low. Regarding the eNanoMapper ontology, a higher coverage was expected as eNanoMapper is considered to provide the full range of terminology needed to describe nanomaterials safety research [64, 65]. The low coverage might be explained by the fact that either the corresponding level of detail of the MIT labeled fields is largely unsupported by the targets or the content of the two target examples differs from the content of the MIT.

To get a more detailed insight into possible reasons for this mismatch, we considered the hierarchical arrangement of the different labeled fields. Almost all labeled fields for grouping of the MIT could be assigned to suitable equivalents within both, the eNanoMapper ontology and the ISA-TAB-Nano standard. Overall, this indicated a similar content of the target schemas as compared to the source schema. However, due to the varying hierarchical concept behind, in the MIT, the labeled fields for grouping contain different subordinate content-bearing labeled fields as compared to the target schemas.

It should be noted that the eNanoMapper ontology also follows a modular approach, comprising six top-level concepts (see Additional file 1: Fig. S1). However, the hierarchical structure of labeled fields imported from external ontologies was adopted unchanged and additional subordinate labeled fields were included. It appears that this fact reduces the claim of an understandable and consistent structure. The degree of specification is also based on a different concept compared to the MIT, which is why labeled fields are not grouped in the same way. Due to the deviating grouping, the identification of the hierarchical position of the upper labeled fields and subordinate labeled fields in the target schema was limited.

In contrast, for ISA-TAB-Nano, the four modules (see Additional file 1: Fig. S2) are organized according to the basic concepts of “Investigation” (project context), “Study” (research unit), “Assay” (analytical measurement) and “Material” (material characterization) to support data entry in a more general way. The hierarchy within ISA-TAB-Nano corresponds more to that of the MIT, although not at the same level of detail. For example, ISA-TAB-Nano does not contain separate modules for a more detailed description of used instruments and their settings (only in the module "Investigation file" in the form of the content-bearing labeled fields "Study assay technology platform" and "Study assay technology type") or for data analysis or statistical evaluation carried out (only listed here in the "Assay file" module in the form of the content-bearing labeled field "Statistic"). It can be stated that the content of the module "Investigation file" roughly corresponds to the MIT module "General information", the module "Study file" to the MIT module "Biological model information", the module "Material" to the MIT module "Material information" and the module "Assay" is like the MIT module "Endpoint readout information".

In total, the one-to-one mapping of labeled fields for grouping corresponds to 24% (8 of 33) with eNanoMapper and 15% (5 of 33) with ISA-TAB-Nano. The overall matching percentage of the remaining content-bearing labeled fields **c**orresponds to 35% (105 of 300) for eNanoMapper and 17% (50 of 300) for ISA-TAB-Nano, respectively.

### Partial mapping

In the first step described above, a one-to-one mapping was performed to find the exact equivalents for the labeled fields of the MIT. In a second step, deviating names or more generally labeled fields (*e.g.,* “protocol” as compared with “dispersion protocol”) were also considered to increase the mapping.

This partial mapping resulted in an increased matching percentage of 77% (231 of 300) for eNanoMapper and 86% (257 of 300) for ISA-TAB-Nano. The higher coverage obtained for ISA-TAB-Nano is primarily caused by the presence of generic content-bearing labeled fields such as “Characteristics”, “Parameter Value Parameter Term”, “Factor Value Factor Term” or “Measured Value Measurement Term'' that can be specified by using content-bearing labeled fields obtained from external ontologies such as the NPO. For specification, the section header “Ontology Source Reference “ is provided within ISA-TAB-Nano, which can be used to enter information on ontologies (“Term Source Name”, Term Source File”, “Term Source Version” and “Term Source Description”) that contain the referenced labeled fields.

**Fig. 3:**
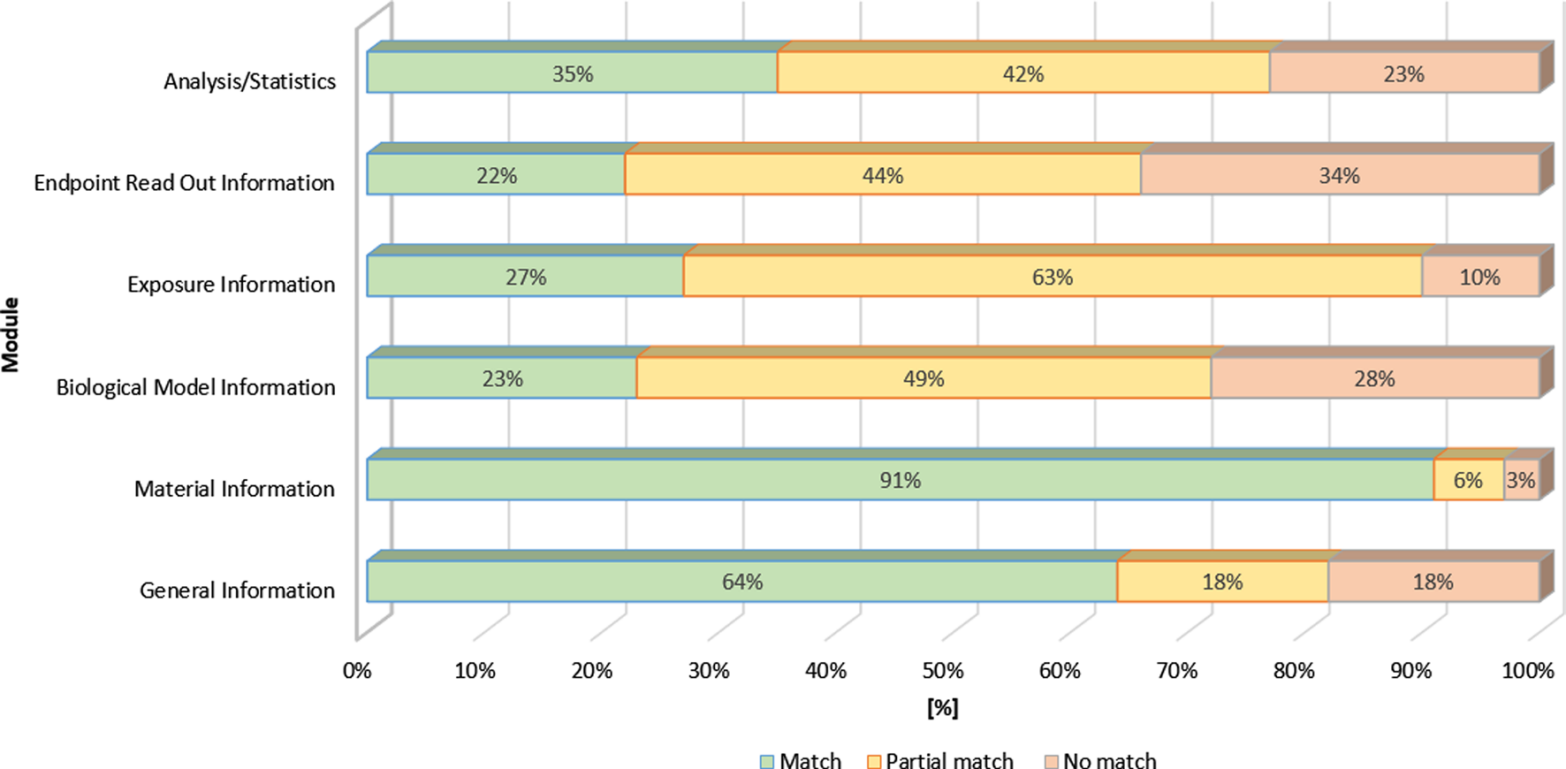
Mapping of the MIT with the eNanoMapper ontology (percentage of one-to-one mapping, total number of content-bearing labeled fields = 300)

Considering the assignment of the content-bearing labeled fields to each module of the MIT (Fig. 3), it was discovered that the eNanoMapper ontology particularly covers the information provided in the MIT modules „Material Information “ (91%, 29 of 32) and „General Information “ (64%, 14 of 22), based on partial mapping. Only equivalents for information on the material design rationale, data access, funding, abbreviations, or supplemental material could not be found in the eNanoMapper ontology. In contrast to the MIT, eNanoMapper offers additional content-bearing labeled fields *e.g.*, to describe different examples for applications of materials (provided within the MIT by the labeled field “Major use”) such as “brightener” or “catalyst” or for the different types of ENMs (provided within the MIT by the labeled field “Compound name”) such as “fullerene”, “nanoclay”, etc. For the remaining four modules “Biological Model Information” (23%, 16 of 69), „Exposure Information “ (27%, 13 of 48), „Endpoint Read Out Information “ (22%, 22 of 98) and „Analysis/Statistics “ (35%, 11 of 31), only a low level of overlap between the eNanoMapper ontology and the MIT was detected (Fig. 3). However, partial mapping of content-bearing labeled field increased the overlap within these modules by approx. 20%—30%. For example, in the eNanoMapper ontology no corresponding content-bearing labeled fields could be found providing background information on the test system or model organism (*e.g.,* phenotype, genetic modification or donor details such as ethnicity, health status or acceptance and quality criteria), license information (*e.g.,* for animal facilities or method and software), details of the cell culture materials used (*e.g.,* type of well plate) and possible assay interferences. Furthermore, in the eNanoMapper ontology, content-bearing labeled fields are missing, which specify the method used (*e.g.,* known uses, applicability, or robustness) and the setting of the instruments (*e.g.,* configuration, magnification, and numerical aperture of the lens). These content-bearing labeled fields are listed within the MIT module „Endpoint Read Out Information “. Compared to the MIT, the eNanoMapper ontology offers concrete examples for guidelines of the OECD (*e.g.,* for toxicokinetics) or assays (*e.g.,* flow cytometry assay) and measurement techniques (*e.g.,* dynamic light scattering (DLS)) relevant for toxicological assessment and biological and physicochemical characterization. The eNanoMapper ontology also includes content-bearing labeled fields for ecosystem types (*e.g.,* from alpine to tropical habitats) and environmental materials (*e.g.,* aerosols such as smoke or solids such as minerals) that are of interest for environmental toxicology or ecotoxicology. Furthermore, specific examples of adverse effects on specific organs or tissues (*e.g.,* eye, skin, or respiratory system) that can be important for the toxicological assessment are also presented.

**Fig. 4:**
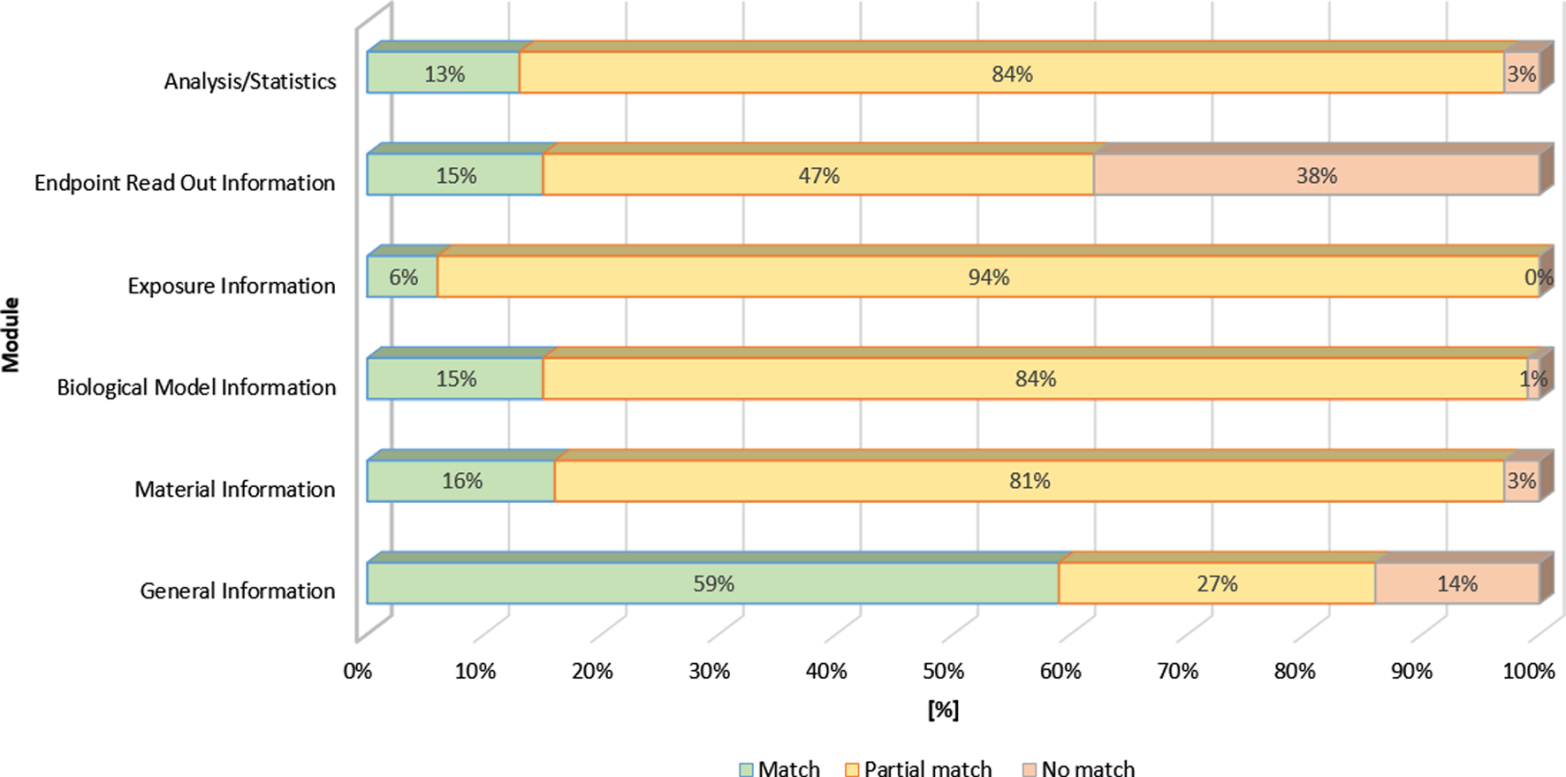
Mapping of the MIT with the ISA-TAB-Nano Format (percentage of one-to-one mapping, total number of content-bearing labeled fields = 300)

With respect to ISA-TAB-Nano, the partial mapping revealed a high overlap (59%, 13 of 22) in the module „General Information “ (Fig. 4). Only for components such as PubMed Central Identifier or abbreviations and definitions, no suitable equivalents were identified. In the modules „Material Information “, „Biological Information “, „Exposure Information “ and „Analysis/Statistics “, the percentage of exact matching was between 6%—16%. Taking partial mapping with generic labeled fields into account (e.g., MIT “dispersion protocol” partial mapping to eNanoMapper “protocol”), the percentage increased significantly („Material Information “ 81% (26 of 32), „Biological Information “ 84% (58 of 69), „Exposure Information “ 94% (45 of 48) and „Analysis/Statistics “ 84% (26 of 31)). The generic labeled fields should offer the possibility to individually annotate experimental investigations and the resulting data using ontology terms. This fact leads to a high level of compatibility with MIT labeled fields, which also implies the lack of more specific content-bearing labeled fields, *e.g.,* for a more precise description of the device information. This is then reflected in the percentage for the module “Endpoint Read out Information'' (15%, 15 of 98), as shown in Fig. 4.

In summary, these results showed that the developed MIT is neither fully covered by the eNanoMapper ontology nor the ISA-TAB-Nano format. Expressed differently, the MIT exhibits a higher level of granularity compared to the considered target schemas. This difference in granularity is due to different levels of detail and covered content of the various approaches. Nevertheless, the two analyzed examples provide a good basis for modelling most of the MIT content. In order to allow for recording of the currently uncovered MIT information by means of the two selected standards, the MIT information would have to be integrated.

It should be noted that the analyzed eNanoMapper ontology describes the various aspects of nanosafety research from a semantic point of view. In contrast, the MIT describes these aspects rather at the metadata level.

To obtain the same level of description, which would be a prerequisite for a semantically correct integration of the MIT content into the eNanoMapper ontology, the MIT would need to be formalized according to an ontology. In this context, interoperability is of crucial importance. Interoperability is generally achieved by using common concepts for knowledge representation, e.g., through the use of a widespread top-level ontology, which is extended by domain-specific entities depending on the application purpose, or by performing mapping to already existing concepts, as has also been shown in the context of this work. A proposal for a conceptualization of an ontology based on the MIT can be found in Additional file 1: Fig. S3. In order to be able to map the MIT completely by the analyzed test cases and to ensure interoperability, it would be necessary to extend the existing ontologies/standards. This could be realized in the future by creating an ontology based on the MIT and integrating concepts from there into eNanoMapper or ISA-TAB Nano that are currently not yet available.

As described above, the compiled MIT could serve as basis for an ontology. It contains concise information about a basic set of metadata and is intended to contribute to increasing the common understanding of their designation and importance in the community. The modular approach enables a context-dependent individualization of the data description to accommodate the interdisciplinary character of nanosafety research. An implementation of descriptions standards derived from the MIT in an ELN-supported system can then contribute to a daily routine of easier (meta)data acquisition in a standardized way throughout the research life cycle. This in turn supports an appreciable increase in quality and quantity, but also a more reliable and traceable re-use of nanosafety research data. To prove the applicability of the MIT in daily research, a case study was carried out to demonstrate the importance of metadata and quality criteria for the development and application of novel non-animal alternative testing methods, which are currently not covered by existing standards and guidelines. Three examples of NAMs including the collection of metadata and quality criteria and their integration into the MIT will be exemplified in the following section.

## New approach methodologies (NAMs) as a use case for structured data collection in nanosafety assessment

The introduction of advanced non-animal alternative test systems in nanosafety research adds further complexity and new challenges to the development of metadata description standards. Non-animal alternative models are being developed that aim to mimic more realistic exposure scenarios and physiological conditions as closely as possible for better in vitro to in vivo extrapolation. Important models were developed to allow for improved risk assessment of ENMs related to inhalation (*e.g.* [66–69]*,*), ingestion [70–72] and dermal absorption [73–75]) as the three relevant routes of exposure. In contrast to conventional in vitro methods, additional parameters and metadata description are necessary for such complex models that aim to mimic the respiratory tract, the intestine, and the skin most realistically. Thus, complementary exercise for precise description of metadata standards is required to ensure robustness and reproducibility for any individual newly developed in vitro model. The implementation of such description standards might accelerate the development of standard procedures on a mode-to-model basis required for early and comprehensive hazard and risk assessment. Here we discuss especially additional required metadata and key parameters based on all six modules of the MIT (General Information, Material Information, Biological Model Information, Exposure Information, Endpoint Read Out Information and Analysis and Statistics) using three non-animal alternative model examples (Additional file 1: Table S2).

### Example 1: air liquid interface (ALI)

The first example is represented by innovative air liquid interface (ALI) systems which have been developed to mimic inhalation and deposition of airborne particles in the respiratory tract [66, 68, 69]. Important intracellular crosstalk mechanisms can be captured by co-culturing different lung cell types to mimic specific regions of the respiratory tract (*e.g*., upper airways, alveoli) and physiological processes of mass transfer to and within the epithelial layers of the lung. Thus, the first key module based on the MIT is the “Biological Model Information”. Besides general cell culture data, such as cell origin (*e.g.*, cell type, donors), cell differentiation and metabolic capacities, ALI-approaches also require model-specific parameters. For instance, air liquid culture conditions on the apical versus the basal side, surfactant or mucus production, tight junctions, cell coverage by fluids, cultivation at submerse vs ALI conditions and ratio of multiple cell type cultures must be reflected by the metadata. Furthermore, in the ALI models, cells are exposed in more realistic conditions via the air. Cells exposed under such simulated exposure of the airways require specific needs for the reporting of metadata especially on the “Exposure Information” which builds the second key module based on the MIT [67]. The generation of the aerosols, as well measurement of their concentration in the generated airflow requires highly sophisticated technology. This includes additionally required metadata description regarding aerosol generation, dilution, volume, flow alignment and particle deposition. Exposure to aerosols is technically challenging because the aerosols need to be generated in an exposure chamber under conditioned flow rate, temperature and humidity and applied to a medium free cell surface. Particle generation itself can be achieved by various approaches including a droplet generator (resuspended particles), combustion engine (*e.g.*, diesel exhaust), or spark ablation generator (nanoparticles). In contrast, under submerged cell culture conditions, ENMs interact with the cell culture medium, and this can markedly affect their physicochemical properties. Regarding the described additional key parameters based on biological model, exposure and dose metrics information modules, ALI systems can build on methods (exposure analyses, modelling) available from “in vivo'' inhalation toxicology research. The third key module based on our example builds the “Endpoint Read Out Information” including additional parameters to describe. epithelial integrity (barrier function) and/or epithelial dysfunction (loss of cell–cell communication).

### Example 2: advanced models of the intestine

The second example is represented by the development of advanced models of the intestine, which represents an entrance organ for which nanosafety research is relatively under-represented as compared with the respiratory tract [70, 72]. The first key module based on the MIT is again the “Biological Model Information”. To mimic realistic exposure scenario, intestinal models include differentiated co-culture cell models to reflect the intestinal mucus barrier, approaches to imitate interactions with the food matrix, digestion-relevant constituents, peristalsis, shear stress and the microbiota as well as more advanced organoid/organ-on-a chip approaches [70, 71]. Outcomes strongly depend on the used intestinal multicellular environment: 2D, 3D systems with or without permeable support systems and the presence of specific cell types, like M cells or goblet cells which increase ENM uptake or can produce and secrete an intestinal mucus layer [71]. Thus, besides general cell culture data, such as cell type, cell differentiation and metabolic capacities, GIT-approaches also require additional model-specific parameters. These parameters include passage number, culturing time, seeding density and ratio, recommended culture conditions like permeable supports and orientation, properties mimicking healthy or inflamed intestine and mucus production verification. The mucus can interact with the ENMs and prevent direct interactions with the epithelium epithelium [76] which is implicated in the second key module: “Exposure Information”. Next to general parameters in this section, model-specific metadata mainly focusing on ENM interactions with dispersing agents as food model to simulate an array of dietary conditions *e.g.*, oil-in-water emulsion [70]. Regarding in vivo conditions, ENMs can be digested by the pH next to salts and proteins in a cell free digestions simulator prior exposure, which changes physicochemical properties like agglomeration size and surface properties of the ENMs and are therefore important to consider as model-specific parameters as well [70]. The third key module based on our example builds the “Endpoint Read Out Information”. The parameter cytokine release can be an indicator for inflammation induced by the ENMs or for validation of stable vs. inflamed co-culture and could also be used for quality control of the biological model.

### Example 3: 3D skin models

The third example is represented by 3D skin models widely used for regulatory purposes as another entrance organ that have become commercially available like EpiSkinTM (EpiSkin Research Institute, Lyon, France), EpiDermTM (MatTech Co., Ashland, MA, USA), and SkinEthicTM (EpiSkin Research Institute) [74, 75]. The skin is composed of three main layers: epidermis, dermis, and subcutaneous tissue. One key endpoint of ENM dermal exposure toxicity studies is the quantification of ENM dose penetration in deeper skin layers. For ENM dermal skin penetration, the type of dispersing agent and the physicochemical properties are likely to play an important role [77] as well as the biological model (*e.g.*, skin model), representing healthy or damaged skin (*e.g.*, UV-B damaged skin) [78] or phototoxicity on the skin model in the presence of UV radiation [75]. Thus, again the described model-specific additional parameters could be stated to correspond to the “Biological Model Information” and “Exposure Information” modules accordingly to the MIT. Skin models furthermore require additional parameter description based on the “Endpoint Read Out Information” MIT module. Irritation, sensitization, and corrosion are important skin related toxicity parameters next to ENM dose penetration. In contrast, general toxicity endpoints like cytotoxicity and cytokine secretion are already stated in the “Endpoint Read Out Information” MIT module [74, 79].

Through any route of exposure, a key requirement for alternative in vitro methods is the relevance and comparability with the anatomical feature being modelled to feed into adverse outcome pathways (AOP) and discover new cellular and molecular mechanisms induced by ENMs [12]. However, the more complex the in vitro models are, the more difficult is the validation and standardization for interlaboratory comparison. Obviously, each of these novel in vitro models require, beyond their validation, detailed parameter descriptions and documentations especially regarding the MIT modules “Material information” (generation and characterization of the ENMs), “Biological model information” and “Exposure information” (*i.e.*, dosimetry). Kämpfer et al*.* suggested recently in this context that the choice of parameters included in a testing strategy for the intestine should preferably be based on the research question to be addressed [71]. This could be transferred to any alternative in vitro model discussed above.

## Conclusion

The derived MIT aims at providing a scientific basis for a modular metadata schema in nanosafety research based on existing standards or guidelines. It is divided into six modules: general information, material information, biological model information, exposure information, endpoint read out information and analysis and statistics. The collected and validated MIT contains more than 300 important parameters for nanosafety research, which will be complemented with additional specific parameters in the future. To explore whether the collected MIT could be mapped by existing open standards or ontologies two test cases, the eNanoMapper Ontology and ISA-TAB-Nano, were selected. The results showed that the developed MIT is neither fully covered by the eNanoMapper ontology nor the ISA-TAB-Nano format caused by the difference in granularity based on the dissimilar concepts of the approaches. A first conceptualization of an ontology based on the MIT was suggested that might contribute to converting the MIT into a machine-readable format. The resulting schema might be used as basis to support structured and modular data acquisition, *e.g.*, by use of ELNs. The use of description standards and quality criteria in nanosafety research is expected to bring crucial improvement of data completeness and reproducibility and will facilitate data comprehensibility and re-usability of future research. In this work we took one first step towards the development of a modular metadata schema in nanosafety, starting from information provided by existing standards and guidelines. New approach methodologies (NAMs) offer new opportunities for improved hazard and risk assessment of ENMs. As illustrated by the case studies, they also bring new challenges regarding the collection of model specific data especially biological model and exposure information. Ideally, data curation strategies should form an a priori component of any investigation or the development of new alternative test methods for nanosafety. Future digitization activities will serve to support such strategies towards an early and structured collection of digital meta(data) as well data re-use. It will be a future challenge to coordinate the variety of guideline development projects and engage stakeholders and research communities to ensure the use of description standards and quality criteria in a harmonized way.

## Supplementary Information

**Additional file 1.** Supplementary materials.** Table S1**. Literature overview on nanoparticle characterizationmethods; Description of the used test cases,** Figures S1 and S2**;** Figure S3**. Conceptualization of an ontologybased on the MIT;** Table S2**. Challenges for NAMs;** Table S3**. Glossary of terms.

**Additional file 2.** Minimum information table.

## Data Availability

All data generated or analyzed are included in this published article and supplementary file. The MIT is available as an.xls file for re-use (Additional file 2).

## Abbreviations

ALI: Air liquid interface
AOP: Adverse outcome pathway
ARRIVE: Animal research: reporting of in vivo experiments
BAO: BioAssay ontology
caNanoLab: NCI’s cancer nanotechnology laboratory
CCLRC: Council for the central laboratory of the research councils
ChEBI: Chemical entities of biological interest
ChEBI: Characterization of nanomaterials in cancer nanotechnology research
CHEMINF: Basic chemical information ontology
CSMD: CCLRC scientific metadata model
DCMI: Dublin core metadata initiative
DLS: Dynamic light scattering
ELN: Electronic lab notebooks
ENM: Engineered nanomaterials
ENVO: Environmental ontology
FAIR: Findability, accessibility, interoperability and reusability
GO: Gene ontology
HTS: High throughput screening
IAO: Information artifact ontology
ISA-Tab: Investigation study assay tabular
ISO: International organization for standardization
JSON: JavaScript object notation
LINCS: Library of integrated network-based cellular signatures
MIRIBEL: Minimum information reporting in bio–nano experimental literature
MIT: Minimum information table
NAMs: New approach methodologies
NBI: Nanomaterial-biological interactions
NCI: National cancer institute
NOAEC: No-observed-adverse-effect concentration
NPO: Nanoparticle ontology
OAE: Ontology of adverse events
OBI: Ontology for biomedical investigation
OBO: Open biological and biomedical ontology
OECD: Organisation for economic co-operation and development
OWL: Web ontology language
PATO: Phenotype and trait ontology
REACH: Registration, evaluation, authorization and restriction of chemicals
SEM: Scanning electron microscope
SOP: Standard operating procedures
TEM: Transmission electron microscopy
TGA: Thermal gravimetric analysis
UBERON: Uber anatomy ontology
URI: Uniform resource identifier
UV–VIS-NIR spectroscopy: Ultra-violet, visible and near infra-red spectroscopy
WPNM: Working party on manufactured nanomaterials
XML: Extensible markup language
