# Supplementary material for: Digital research data: from analysis of existing standards to a scientific foundation for a modular metadata schema in nanosafety
Source: Part Fibre Toxicol. 2022 Jan 5;19:1. doi: 10.1186/s12989-021-00442-x (PMC8728981; doi:10.1186/s12989-021-00442-x)
Supplement: Supplementary file 1 — Additional file 1. Supplementary materials. Table S1. Literature overview on nanoparticle characterizationmethods; Description of the used test cases, Figures S1 and S2; Figure S3. Conceptualization of an ontologybased on the MIT; Table S2. Challenges for NAMs; Table S3. Glossary of terms. [file 12989_2021_442_MOESM1_ESM.docx]

**Supplementary file**

**Digital Research Data: From Analysis of Existing Standards to a Scientific Foundation for a Modular Metadata Schema in Nanosafety**

Linda Elberskirch^1†^, Kunigunde Binder^2†^, Norbert Riefler^3^, Adriana Sofranko^4^, Julia Liebing^5^, Christian Bonatto Minella^2^, Lutz Mädler^3^, Matthias Razum^2^, Christoph van Thriel^5^, Klaus Unfried^4^, Roel P.F. Schins^4^, Annette Kraegeloh^1*^

^1^INM - Leibniz Institute for New Materials, Campus D2 2, 66123 Saarbrücken

^2^FIZ Karlsruhe – Leibniz Institute for Information Infrastructure, Hermann-von-Helmholtz-Platz 1, 76133 Eggenstein-Leopoldshafen

^3^IWT - Leibniz-Institut für Werkstofforientierte Technologien, Badgasteiner Str. 3, 28359 Bremen

^4^IUF - Leibniz Research Institute for Environmental Medicine, Auf'm Hennekamp 50, 40225 Düsseldorf

^5^IfADo - Leibniz Research Centre for Working Environment and Human Factors, Ardeystraße 67, 44139 Dortmund

*Corresponding author

† Contributed equally

**Content**

| **Name** | **Page** |
| --- | --- |
| Table S1: Literature Overview on Nanoparticle Characterization Methods | 1 - 15 |
| **Description of the used test cases:** | 16 - 21 |
| Test case 1: eNanoMapper Ontology | 16-17 |
| - Figure S1: Overview of concepts within the eNanoMapper ontology | 18 |
| - Test case 2: ISA-TAB-Nano | 19 |
| - Figure S2: Representation of the ISA-TAB Nano specification structure | 19 |
| Figure S3: Overview conceptualization of an ontology based on the MIT | 22 - 23 |
| Table S2: Challenges for new approach methodologies (NAMs) | 24 - 25 |
| Table S3: Glossary of terms used in the present article | 26 - 29 |

**Table S1: Literature Overview on Nanoparticle Characterization Methods**

## Introduction

There are no physical mechanisms for a comprehensive characterization of nanoparticles. Only a combination of different methods permits identification, quantification, and characterization [Miernicki et al. 2019]. 40 methods are listed in the following and in the accompanying table sheet. Nearly half of them are inverse methods where the effects are measured and the causing parameters have to be calculated with complex algorithms, which often limit the quality of the results. In contrast, spectroscopic methods like UV-vis or FTIR are direct methods and deliver a wealth of information, useful to observe parameter differences during an investigation. However, this information has often no direct physical relation and can be used only as relative measure based on comparisons. So far as issues about a particular measurement method are known, they will be mentioned in the table sheet.

## Short Literature Review

In a recent perspective article, [Faria et al. 2018] gave good reasons for what they called Minimum Information Reporting in Bio-Nano Experimental Literature (MIRIBEL), which requires minimum information to specify interaction results between nanomaterials and biological systems in three categories: Material characterization, biological characterization, and experimental protocol details.

The biophysical interactions at the nano-bio interface are described by [Nel et al. 2009], with zeta potential, rigid core size and hydrophobicity as main parameter concerning nanoparticle biocompatibility.

Main emphasis on the three principles of transport, surface, and material in nanotoxicology are discussed by [Krug et al. 2011]. The transport principle requires nanoparticle characterization in wet and dry media and the surface and material principle emphasize surface characterizing methods.

An overview of measurement techniques of many physical-chemical properties is presented by [Rasmussen et al. 2016]. However, this paper shows inconsistencies and uncertainties like, e.g., how Dynamic Light Scattering (DLS) can be used to infer crystallite size of nanoparticles, or why they split crystall structure in crystalline phase and crystallite size. Furthermore, there are no references related to each entry in their tables. In contrast, the review of [Mourdikoudis et al. 2018] gives a thorough list of reviews about each measurement technique for characterizing nanoparticles. In a protocol paper of [DeLoid et al. 2017], there is a compilation list (but still not comprehensive) of other characterisation methods. A less extensive but very clear and thorough discussion about useful techniques for the size measurement of nanoparticles is given in the review by [Linsinger et al. 2012]. Comparison of different particle sizing methods on, e.g., bimodal particle size distribution is presented by [Babick et al. 2016] as well as by [Tiede et al. 2008].

The following citations provide additional information about characterization methods as a supplement to the overview of Table 2 in [Rasmussen et al. 2016]. Some parameters suggested in [Rasmussen et al. 2016] are not covered by standard measurement techniques and, therefore, will be mentioned in the following. Most of the citated papers and books are included in the tabular overview.

AFM can be used to characterize size and shape [Couteau et al. 2011] as well as surface interaction of nanoparticles with cells [Pyrgiotakis et al. 2014]. Additionally, AFM is used to measure the mechanical strength of aggregates and aggomerates [Salameh 2015]. Methods to measure the density of NPs are presented in [Carney et al. 2003], [Park et al. 2004], [Liao et al. 2018] and [Naito et al. 2018]. Both, EDX and EELS as a supplement to TEM, delivers information about the surface chemistry of nanoparticles [Hagendorfer et al. 2011] as well as the irradiation of samples with X-rays in XPS [Tantra 2016]. SAXS methods are reviewed by [Li et al. 2016]. The crystallite size is important for the chemical stability of NPs shown by [Banham et al. 2014]. BET measurements enable the estimation of the surface of nanoparticles in relation to their weight (i.e., to their volume). This information can be used to get a mean primary particle diameter [Pokhrel et al. 2010]. The BET method is compared in detail with SEM in [Wohlleben et al. 2017] and it is stated that the measurement of the volume-specific surface area via BET is a reliable method for nanoparticle characterization. Investigation of the surface chemistry of nanoparticles can be performed with NMR [Baalousha et al. 2015], [Bhagyaraj et al. 2018], [Tantra 2016] and [Thomas et al. 2017].

The attenuation of ultrasonic waves over a frequency spectrum delivers information to get the particle size distribution of nanoparticles [Wang et al. 2009], [Dukhin 2002] and [Povey 1997]. ICP-MS or ICP-OES can be used to measure size of nanoparticles and their dissolution within the media, see [Tantra 2016], [Vencalek et al. 2016], [Zook et al. 2011] and [Hagendorfer et al. 2011].

Direct methods of characterizing the hydrophobicity of nanoparticles are treated by [Valsesia et al. 2018], [Cao et al. 2019] and [Xiao et al. 2012]. Cyclic voltametry [Thomas et al. 2017], [Kumar et al. 2018] is used to characterize electrochemical properties on gold nanoparticles [Brust et al. 2012] or the surface states of modified TiO_2_ nanoparticles [de la Garza et al. 2006]. UV-vis is used to measure the size, concentration and agglomeration of nanoparticles in [Haiss et al. 2007], [Zook et al. 2011] and [Bian et al. 2011]. Additionally, UV-vis can be used to deliver the dispersity and/or solubility of nanoparticles shown in [Motomizu et al. 1989]. Turbidimetry or nephelometry is compared with UV-vis measurement methods by [Kozin et al. 2017]. FTIR is described by [Djurisic et al. 2015] and [Thomas et al. 2017]. DLS is reviewed by [Xu 2015]. The electrophoretic mobility is often measured within a DLS device, see [Margus et al. 2016]. CLS/DCS is described in [Scott et al. 2005]. A direct comparison between DLS and CLS for nanoparticles in the size range of 30--50 nm is performed in [Braun et al. 2011] and in [Anderson et al. 2013]. A comparison of DLS and PTA is presented in [Filipe et al. 2010]. Characterization of the impact of nanoparticles on cells can be performed by Flow Cytometry [Reineke 2012]. Dissolution of nanoparticles is reviewed by [Misra et al. 2012] and is investigated in [Zook et al. 2011] and [Vencalek et al. 2016] with an ICP-MS, while [Odzak et al. 2015] compares different separation mechanisms. A review about Flow Field Flow Fractionation is presented by [Baalousha et al. 2011], [Hagendorfer et al. 2011] and [Wahlund 2013]. The use of electrophoresis to characterize nanoparticles are described by [Hanauer et al. 2007] and in particular for Capillary Electrophoresis (CE) by [Brambilla et al. 2010]. The measurement of size of particles or macromolecules with static light scattering detected under different angles is described by [Dick et al. 2007], [Mehn et al. 2017] and [Xie et al. 2002]. Statistical information about the size of nanoparticles is given by LIBS [Baalousha et al. 2011]. The fluorescence spectrum is characteristic for molecules and can be measured by LIF [Brambilla et al. 2010], [Ban et al. 2013] as a supplement to CE. Raman spectroscopy delivers information about the crystal structure of nanoparticles as well as surface chemicals [Kode et al. 2012], [Mansfield et al. 2017]. TGA is another method to get information about the surface chemistry and hydrophobicity [Wang et al. 2011]. Other methods for hydrophobicity measurements are described by [Cao et al. 2019], [Valsesia et al. 2018] and [Xiao and Wiesner 2012].

Field Flow Fractionation (FFF) is a separation method which is often combined with other measurement methods to infer size distribution of nanoparticles [Baalousha et al. 2011], [Hagendorfer et al. 2011], [Wahlund 2013]. Membrane filtration is, e.g., described by [Gaborski et al. 2010] or [Tsao et al. 2011]. DMA is a fractionation method but useable to yield size distributions and particle density as well [Lenggoro et al. 2002]. Investigation of the formation of radicals in biological environments are performed with potentiometry [Ibupoto et al. 2013] and [Skoog et al. 2004]. The degradability of prebiotic material is characterized by ion exchange chromatography (IC) [Riviere et al. 2013]. The same method is used for investigations of biogeochemical processes in [Smith et al. 2002]. Bioaccumulation is characterized by Liquid Scintillation Counting (LSC) [Avanasi et al. 2014], by High Pressure Liquid Chromatography (HPLC) or Liquid Chromatography Mass Spectroscopy (LC-MS) [Petersen et al. 2019], and by Inductively Coupled Plasma Mass Spectroscopy (IC-PMS), see above for references.

## References

Anderson, W., Kozak, D., Coleman, V. A., Jämting, Å. K. & Trau, M. (2013). A comparative study of submicron particle sizing platforms: Accuracy, precision and resolution analysis of polydisperse particle size distributions. *JCIS*, 405, 322-330.

Avanasi, R., Jackson, W. A., Sherwin, B., Mudge, J. F. & Anderson, T. A. (2014). C60 Fullerene Soil Sorption, Biodegradation, and Plant Uptake. *Environ. Sci. Technol.*, 48, 2792-2797.

Baalousha, M. & Lead, J. R. (2015). *Characterization of Nanomaterials in Complex Environmental and Biological Media*. : Elsevier.

Baalousha, M., Stolpe, B. & Lead, J. (2011). Flow field-flow fractionation for the analysis and characterization of natural colloids and manufactured nanoparticles in environmental systems: A critical review. *J. Chromatography A*, 1218, 4078-4103.

Babick, F., Mielke, J., Wohlleben, W., Weigel, S. & Hodoroaba, V.-D. (2016). How reliably can a material be classified as a nanomaterial? Available particle-sizing techniques at work. *J. Nanopart. Res.*, 18, 158-1-40.

Ban, E. & Song, E. J. (2013). Recent developments and applications of capillary electrophoresis with laser-induced fluorescence detection in biological samples. *J. Chromatography B*, 929, 180-186.

Banham, D., Ye, S., Cheng, T., Knights, S., Stewart, S. M., Wilson, M. & Garzon, F. (2014). Effect of CeOx Crystallite Size on the Chemical Stability of CeOx Nanoparticles. *J. Electrochem. Soc.*, 161, F1075-F1080.

Bhagyaraj, S. M., Oluwafemi, O. S., Kalarikkal, N. & Thomas, S. (2018). *Characterization of Nanomaterials. Advances and Key Technologies*. : Elsevier.

Bian, S.-W., Mudunkotuwa, I. A., Rupasinghe, T. & Grassian, V. H. (2011). Aggregation and Dissolution of 4 nm ZnO Nanoparticles in Aqueous Environments: Influence of pH, Ionic Strength, Size, and Adsorption of Humic Acid. *Langmuir*, 27, 6059-6068.

Brambilla, D., Verpillot, R., Taverna, M., Kimpe, L. D., Nicolas, B. L. D. J., Canovi, M., Gobbi, M., Mantegazza, F., Salmona, M., Nicolas, V., Scheper, W., Couvreur, P. & Andrieux, K. (2010). New Method Based on Capillary Electrophoresis with Laser-Induced Fluorescence Detection (CE-LIF) to Monitor Interaction between Nanoparticles and the Amyloid- Peptide. *Anal. Chem.*, 82, 10083-10089.

Braun, A., Couteau, O., Franks, K., Kestens, V., Roebben, G., Lamberty, A. & Linsinger, T. (2011). Validation of dynamic light scattering and centrifugal liquid sedimentation methods for nanoparticle characterisation. *Adv. Powder Techn.*, 22, 766-770.

Brust, M. & Gordillo, G. J. (2012). Electrocatalytic Hydrogen Redox Chemistry on Gold Nanoparticles. *J. Am. Chem. Soc.*, 134, 3318-3321.

Cao, Z., Tsai, S. N. & Zuo, Y. Y. (2019). An Optical Method for Quantitatively Determining the Surface Free Energy of Micro- and Nanoparticles. *Anal. Chem.*, 91, 12819-12826.

Carney, R. S. & Schottland, J. C. (2003). Theory of total-internal-reflection tomography. *J. Opt. Soc. Am. A*, 20, 542-547.

Couteau, O. & Roebben, G. (2011). Measurement of the size of spherical nanoparticles by means of atomic force microscopy. *Meas. Sci. Technol.*, 22, 065101-1-8.

DeLoid, G. M., Cohen, J. M., Pyrgiotakis, G. & Demokritou, P. (2017). Preparation, characterization, and in vitro dosimetry of dispersed, engineered nanomaterials. *Nature Protocols*, 12, 355-371.

Dick, W. D., Ziemann, P. J. & McMurry, P. H. (2007). Multiangle Light-Scattering Measurements of Refractive Index of Submicron Atmospheric Particles. *Aerosol Sci. Technol.*, 41, 549-569.

Djurisic, A. B., Leung, Y. H., Ng, A. M. C., Xu, X. Y., Lee, P. K. H., Degger, N. & Wu, R. S. S. (2015). Toxicity of Metal Oxide Nanoparticles: Mechanisms, Characterization, and Avoiding Experimental Artefacts. *Small*, 11, 26-44.

Dukhin, A. (2002). *Utrasound for characterizing colloids*. : Elsevier.

Faria, M., Björnmalm, M., Thurecht, K. J., Kent, S. J., Parton, R. G., Kavallaris, M., Johnston, A. P. R., Gooding, J. J., Corrie, S. R., Boyd, B. J., Thordarson, P., Whittaker, A. K., Stevens, M. M., Prestidge, C. A., Porter, C. J. H., Parak, W. J., Davis, T. P., Crampin, E. J. & Caruso, F. (2018). Minimum information reporting in bio-nano experimental literature. *Nature Nanotechnology*, 13, 777-785.

Filipe, V., Hawe, A. & Jiskoot, W. (2010). Critical Evaluation of Nanoparticle Tracking Analysis (NTA) by NanoSight for the Measurement of Nanoparticles and Protein Aggregates. *Pharm. Res.*, 27, 796-810.

Gaborski, T. R., Snyder, J. L., Striemer, C. C., Fang, D. Z., Hoffman, M., Fauchet, P. M. & McGrath, J. L. (2010). High-Performance Separation of Nanoparticles with Ultrathin Porous Nanocrystalline Silicon Membranes. *ACS Nano*, 11, 6973-6981.

de la Garza, L., Saponjic, Z. V., Dimitrijevic, N. M., Thurnauer, M. C. & Rajh, T. (2006). Surface States of Titanium Dioxide Nanoparticles Modified with Enediol Ligands. *J. Phys. Chem. B*, 110, 680-686.

Hagendorfer, H., Kaegi, R., Traber, J., Mertens, S. F., Scherrers, R., Ludwig, C. & Ulrich, A. (2011). Application of an asymmetric flow field flow fractionation multi-detector approach for metallic engineered nanoparticle characterization – Prospects and limitations demonstrated on Au nanoparticles. *Analytical Chimca Acta*, 706, 367-378.

Haiss, W., Thanh, N. T. K., Aveyard, J. & Fernig, D. G. (2007). Determination of Size and Concentration of Gold Nanoparticles from UV-Vis Spectra. *Anal. Chem.*, 79, 4215-4221.

Hanauer, M., Pierrat, S., Zins, I., Lotz, A. & Sönnichsen, C. (2007). Separation of Nanoparticles by Gel Electrophoresis According to Size and Shape. *Nano Lett.*, 7, 2881-2885.

Ibupoto, Z. H., Khun, K. & Willander, M. (2013). A Selective Iodide Ion Sensor Electrode Based on Functionalized ZnO Nanotubes. *Sensors*, 13, 1984-1997.

Kode, K., Shachaf, C., Elchuri, S., Nolanb, G. & Paik, D. S. (2012). Raman labeled nanoparticles: characterization of variability and improved method for unmixing. *J. Raman Spectrosc.*, 43, 895-905.

Kozin, P. & von der Kammer, F. (2017). Clarification of methodical questions regarding the investigation of nanomaterials in the environment Development of a decision support tool for the investigation of nanomaterial’s environmental behaviour based on dispersion behaviour and dissolution in relation to various environmental parameters. *Umweltbundesamt*, 108, 1-65.

Krug, H. F. & Wick, P. (2011). Nanotoxicology: An Interdisciplinary Challenge. *Angew. Chem. Int. Ed.*, 50, 1260-1278.

Kumar, V., Dasgupta, N. & Ranjan, S. (E. (2018). *Nanotoxicology. Toxicity Evaluation, Risk Assessment and Management*. : CRC.

Lenggoro, I. W., Xia, B., Okuyama, K. & de la Mora, J. F. (2002). Sizing of Colloidal Nanoparticles by Electrospray and Differential Mobility Analyzer Methods. *Langmuir*, 18, 4584-4591.

Li, T., Senesi, A. J. & Lee, B. (2016). Small Angle X‑ray Scattering for Nanoparticle Research. *Chem. Rev.*, 116, 11128-11180.

Liao, B.-X., Tseng, N.-C. & Tsai, C.-J. (2018). The accuracy of the aerosol particle mass analyzer for nanoparticle classification. *Aerosol Sci. Technol.*, 52, 19-29.

Linsinger, T., Roebben, G., Gilliland, D., Calzolai, L., Rossi, F., Gibson, N. & Klein, C. (2012). Requirements on measurements for the implementation of the European Commission definition of the term nanomaterial. *JRC Reference Report*, , 1-52.

Mansfield, E., Kaiser, D. L., Fujita, D. & Voorde, M. V. d. (2017). *Metrology and Standardization for Nanotechnology. Protocols and Industrial Innovations*. : Wiley-VCH.

Margus, M., Milanovic, I. & Ciglenecki, I. (2016). Voltammetric, dynamic light scattering (DLS) and electrophoretic mobility characterization of FeS nanoparticles (NPs) in different electrolyte solutions. *J Solid State Electrochem.*, 20, 2981-2989.

Mehn, D., Caputo, F., Rösslein, M., Calzolai, L., Saint-Antonin, F., Courant, T., Wick, P. & Gilliland, D. (2017). Larger or more? Nanoparticle characterisation methods for recognition of dimers. *RSC Adv.*, 7, 27747-27754.

Miernicki, M., Hofmann, T., Eisenberger, I., von der Kammer, F. & Praetorius, A. (2019). Legal and practical challenges in classifying nanomaterials according to regulatory definitions. *nature nanotechn.*, 14, 208-216.

Misra, S. K., Dybowska, A., Berhanu, D., Luoma, S. N. & Valsami-Jones, E. (2012). The complexity of nanoparticle dissolution and its importance in nanotoxicological studies. *Sci. Total Environ.*, 438, 225-232.

Motomizu, S., Oshima, M. & Ikegami, T. (1989). Flotation Extraction with Molybdate and and Spectrophotometric Cationic Dyes. *Analytical Science*, 5, 767-769.

Mourdikoudis, S., Pallares, R. M. & Thanh, N. T. K. (2018). Characterization techniques for nanoparticles: comparison and complementarity upon studying nanoparticle properties. *Nanoscale*, 10, 12871-12934.

Naito, M., Yokoyama, T., Hosokawa, K. & Nogi, K. (E. (2018). *Nanoparticle Technology Handbook*. : Elsevier.

Nel, A. E., Mädler, L., Velegol, D., Xia, T., Hoek, E. M. V., Somasundarana, P., Klaessig, F., Castranova, V. & Thompson, M. (2009). Understanding biophysicochemical interactions at the nano-bio interface. *Nat. Mater.*, 8, 543-557.

Odzak, N., Kistler, D., Behra, R. & Sigg, L. (2015). Dissolution of metal and metal oxide nanoparticles in aqueous media. *Environmental Pollution*, 191, 132-138.

Park, K., Kittelson, D. B., Zachariah, M. R. & McMurry, P. H. (2004). Measurement of inherent material density of nanoparticle agglomerates. *J. Nanopart. Res.*, 6, 267-272.

Petersen, E. J., Mortimer, M., Burgess, R. M., Hanna, R. H. S., Ho, K. T., Johnson, M., Loureiro, S., Selck, H., Scott-Fordsmand, J. J., Spurgeon, D., Unrine, J., van den Brink, N. W., Wang, Y., White, J. & Holden, P. (2019). Strategies for robust and accurate experimental approaches to quantify nanomaterial bioaccumulation across a broad range of organisms. *Environ. Sci.: Nano*, 6, 1619-1656.

Pokhrel, S., Birkenstock, J., Schowalter, M., Rosenauer, A. & Mädler, L. (2010). Growth of Ultrafine Single Crystalline WO3 Nanoparticles Using Flame Spray Pyrolysis. *Cryst. Growth Design*, 11, 632-639.

Povey, M. J. W. (1997). *Ultrasonic Techniques for Fluids Characterization*. : Academic Press.

Pyrgiotakis, G., Blattmann, C. O. & Demokritou, P. (2014). Real-Time Nanoparticle−Cell Interactions in Physiological Media by Atomic Force Microscopy. *ACS Sustainable Chem. Eng.*, 2, 1681-1690.

Rasmussen, K., Gonzalez, M., Kearns, P., Sintes, J. R., Rossi, F. & Sayre, P. (2016). Review of achievements of the OECD Working Party on Manufactured Nanomaterials' Testing and Assessment Programme. From exploratory testing to test guidelines. *Regul. Toxicol. Pharmacol.*, 74, 147-160.

Reineke, J. (E. (2012). *Nanotoxicity*. : Humana Press.

Riviere, A., Eeltink, S., Pierlot, C., Balzarini, T., Moens, F., Selak, M. & Vuyst, L. D. (2013). Development of an Ion-Exchange Chromatography Method for Monitoring the Degradation of Prebiotic Arabinoxylan- Oligosaccharides in a Complex Fermentation Medium. *Anal. Chem*, 85, 4982-4990.

Salameh, S. (2015). Contact behaviour of metal oxide nanoparticle aggregates. *Dissertation*, .

Scott, D. J., Harding, S. E. & Rowe, A. J. (2005). *Analytical Ultracentrifugation. Techniques and Methods*. : RSC Publishing.

Skoog, D., West, D. M., Holler, F. J. & Crouch, S. R. (2004). *Fundamentals of Analytical Chemistry*. : Thomson.

Smith, E., Davison, W. & Hamilton-Taylor, J. (2002). Methods for preparing synthetic freshwaters. *Water Res.*, 36, 1286-1296.

Tantra, R. (2016). *Nanomaterial Characterization. An Introduction*. : Wiley.

Thomas, S., Thomas, R., Zachariah, A. K. & Mishra, R. K. (2017). *Thermal and Rheological Measurement Techniques for Nanomaterials Characterization*. : Elsevier.

Tiede, K., Boxall, A. B., Tear, S. P., Lewis, J., David, H. & Hassellöv, M. (2008). REVIEW: Detection and characterization of engineered nanoparticles in food and the environment. *Food Addit. Contam.*, 25, 795-821.

Tsao, T. M., Chen, Y. M. & Wang, M. K. (2011). Origin, separation and identification of environmental nanoparticles: a review. *J. Environ. Monit.*, 13, 1156-1163.

Valsesia, A., Desmet, C., Ojea-Jimenez, I., Oddo, A., Capomaccio, R., Rossi, F. & Colpo, P. (2018). Direct quantification of nanoparticle surface hydrophobicity. *Communication Chem.*, 53, 1-11.

Vencalek, B. E., Laughton, S. N., Spielman-Sun, E., Rodrigues, S. M., Unrine, J. M., Lowry, G. V. & Gregory, K. B. (2016). In Situ Measurement of CuO and Cu(OH)2 Nanoparticle Dissolution Rates in Quiescent Freshwater Mesocosms. *Environ. Sci. Technol. Lett.*, 3, 375-380.

Wahlund, K.-G. (2013). Flow field-flow fractionation: Critical overview. *J. Chromatography A*, 1287, 97-112.

Wang, C., Mao, H., Wang, C. & Fu, S. (2011). Dispersibility and Hydrophobicity Analysis of Titanium Dioxide Nanoparticles Grafted with Silane Coupling Agent. *Ind. Eng. Chem. Res.*, 50, 11930-11934.

Wang, X. Z., Liu, L., Li, R. F., Tweedie, R. J., Primrose, K., Corbett, J. & McNeil-Watson, F. K. (2009). Online characterisation of nanoparticle suspensions using dynamic light scattering, ultrasound spectroscopy and process tomography. *Chem. Eng. Res. Des.*, 87, 874-884.

Wohlleben, W., Mielke, J., Bianchin, A., Ghanem, A., Freiberger, H., Rauscher, H., Gemeinert, M. & Hodoroaba, V.-D. (2017). Reliable nanomaterial classification of powders using the volume-specific surface area method. *J. Nanopart. Res.*, 19, 61-1-16.

Xiao, Y. & Wiesner, M. R. (2012). Characterization of surface hydrophobicity of engineered nanoparticles. *J. Hazardous Materials*, 215-216, 146-151.

Xie, T., Penelle, J. & Verraver, M. (2002). Experimental investigation on the reliability of routine SEC–MALLS for the determination of absolute molecular weights in the oligomeric range. *Polymer*, 43, 3973-3977.

Xu, R. (2015). Light scattering: A review of particle characterization applications. *Particuology*, 18, 11-21.

Zook, J. M., Long, S. E., Cleveland, D., Geronimo, C. L. A. & MacCuspie, R. I. (2011). Measuring silver nanoparticle dissolution in complex biological and environmental matrices using UV–visible absorbance. *Anal. Bioanal. Chem.*, 401, 1993-2002.

**Description of the used test cases**

**Test case 1: eNanoMapper Ontology**

The eNanoMapper Ontology (available at https://github.com/enanomapper/ontologies or https://bioportal.bioontology.org/ontologies/ENM/) was developed within the framework of the EU FP7 eNanoMapper project (www.enanomapper.net) for the annotation of engineered ENMs, including comprehensive information on their hazard-related properties, e.g., physicochemical properties, toxicological and environmental impact as well as relevant experimental conditions and procedures [1]. The eNanoMapper ontology is based on several existing open ontologies with relevance in the field of nanosafety. Amongst others, these include the Basic Chemical Information Ontology (CHEMINF), the Chemical Entities of Biological Interest (ChEBI), the Gene Ontology (GO) and the Ontology for Biomedical Investigation (OBI) from the collection of the Open Biological and Biomedical Ontology (OBO) Foundry. The OBO consortium is dedicated to the creation and maintenance of ontologies in the field of biomedicine and has developed a set of principles for ontology development [2]. The extraction and compilation of subsets of external ontology content was done automatically using an open-source tool called "Slimmer" (https://github.com/enanomapper/slimmer/), which was specifically created for this purpose. eNanoMapper in the current version 7.2 (05/15/2021) consists of 11,974 classes and is expressed in the Web Ontology Language (OWL). Moreover, an RDF/XML syntax-based version is also available. As shown in Fig. 3, eNanoMapper describes the knowledge domain of nanomaterial safety using six **top-level classes**:

**Anatomical entity:** this new class introduced with the latest version integrates the “Organ” class from the Uber Anatomy Ontology (UBERON), which covers entities that are classified according to anatomical criteria such as structure, function, and lineage of development in organisms [3,4].

**Disposition:** the disposition class is characterised by a property-based (*e.g.* surface properties) as well as application-based (*e.g.* application as an antioxidant, diluent, etc.) information classification and mainly contains concepts from the NanoParticle Ontology (NPO), which is an ontology that represents the knowledge underlying the description, preparation, and characterisation of nanomaterials in cancer nanotechnology research [5,6], and the ChEBI, an ontology of molecular entities of biological interest that focuses on small chemical compounds [7,8].

**Information content entity:** this class represents a mixture of descriptive (e.g., citation, name, version, etc.) and general information on the assay (e.g., assay title, research institute, protocol, related data etc.), as well as experimental parameters (*e.g.,* experimental, or pharmacokinetic factors, etc.) and endpoints (*e.g.,* biological, physicochemical, or toxicological), including an entity to describe adverse outcomes pathways. To classify all this information, terms from the BioAssay Ontology (BAO; encompasses knowledge of biological screening assays and their results including high-throughput screening data (HTS)) [9,10], the Information Artifact Ontology (IAO; an ontology about information entities) [11,12], and the Adverse Outcome Pathway Ontology (AOP) [13] were adopted.

**Material entity:** the material entity class includes details of experimental components for biological and environmental characterization such as examined nanoparticle types, model organism, instruments (*e.g.,* microscope, photometer, etc.) and other materials used. For their categorisation, classes from the ChEBI, the NPO and the environmental ontology (ENVO) are re-used. The community driven ENVO is composed of terms that describe environmental entities of all kinds, from microscopic to intergalactic scales [14,15].

**Process:** this class is made up of different assay types and measurement techniques relevant to the characterization of ENMs as well as details on the process of ENM synthesis (macroscopic and microscopic), which are provided by the subclass „synthesis part“ of the NPO. The “assay” subclass, which was migrated from OBI, is used to describe the experimental methods. The OBI serves to annotate biological and clinical investigations, including the study design, the protocols and instruments used, the data generated, and the types of analysis performed on them [16,17]. In addition, known safety information about ENM is picked up by the subclass „adverse event“ which is borrowed from the Ontology of Adverse Events (OAE), a community-driven ontology for standardizing and integrating data on biomedical adverse events (*e.g.,* vaccine and drug adverse events) and to aid computer-assisted reasoning [18,19].

**Quality:** the quality class classifies the different types of ENM according to their properties (*e.g.,* physical state, size, solubility, stability, etc.) constituency and shape. To describe these properties, concepts from the CHEMINF containing terms about chemical entities [20,21], NPO and the Phenotype and Trait Ontology (PATO) are incorporated [22].

**Figure S1:** **Overview of concepts within the eNanoMapper ontology** (integrated from external ontologies and manually added). Re-used ontologies: **aop**: Adverse Outcome Pathway Ontology; **bao**: BioAssay Ontology; **bfo**: Basic Formal Ontology; **ccont**:Cell Culture Ontology; **chebi**:Chemical Entities of Biological Interest; **cheminf**: Chemical Information Ontology; **chmo**: Chemical Methods Ontology; **efo**: Experimental Factor Ontology; **enm**: eNanoMapper Ontology; **envo**: Environment Ontology; **evs**: Enterprise Vocabulary Service Thesaurus; **fix**: Physico-chemical methods and properties; **go**: Gene Ontology; **iao**: Information Artifact Ontology; **ncit**: National Cancer Institute Thesaurus; **npo**: Nanoparticle Ontology; **oae**: Ontology of Adverse Events; **obcs**: Ontology of Biological and Clinical Statistics; **obi**: Ontology for Biomedical Investigation; **pato**: Phenotypic quality Ontology; **stato**: The Statistical Methods Ontology; **uberon**: Uber-anatomy Ontology; **uo**: Unit of Measurements Ontology

**Test case 2: ISA-TAB-Nano**

ISA-TAB-Nano is an extension of the Investigation Study Assay Tabular (ISA-Tab) standard, a tab-delimited format specification based on the ISA Abstract Model for capturing experimental metadata of life, environmental and biomedical sciences [23,24]. In contrast to the eNanoMapper ontology, ISA-TAB-Nano is a spreadsheet-based format that was created in 2012 as part of an initiative of the Nanotechnology Working Group from the National Cancer Institute (NCI). The aim was to develop an adequate solution that enabled the data import/export related to nanomaterials and their characterization together with corresponding information (*e.g.,* raw/derived data, image files or protocols) between researchers as well as to/from nanotechnological frameworks, *e.g.,* the NCI’s cancer Nanotechnology Laboratory (caNanoLab) portal or the Nanomaterial-Biological Interactions (NBI) knowledge base.

The latest version corresponds to the 1.3-Version (March 2020). As shown in Figure S2 ISA-TAB-Nano consists of four spreadsheet-based files. The three core entities „Investigation File“, „Study File“ and „Assay File“ were adapted from the recommended ISA-Tab format. The “Investigation File” helps to describe the overall investigation including the corresponding studies and assays but also to collect reference information on them, e.g., with respect to material product sheets, protocols, or relevant publications. The “Study File” instead, contains information about the source, experimental procedure, and properties of the examined subject (biological specimen like cell line, animal, etc.). The “Assay File” records the protocol parameters and factors (e.g., concentration, temperature, media, solvent, etc.) associated with each assay and provides reference to experimental output including measurements, instrumentation as well as derived and further data types (e.g., 3D structure file). The material entity („Material File“) was newly introduced to capture information about the composition and properties of ENMs [25,26].


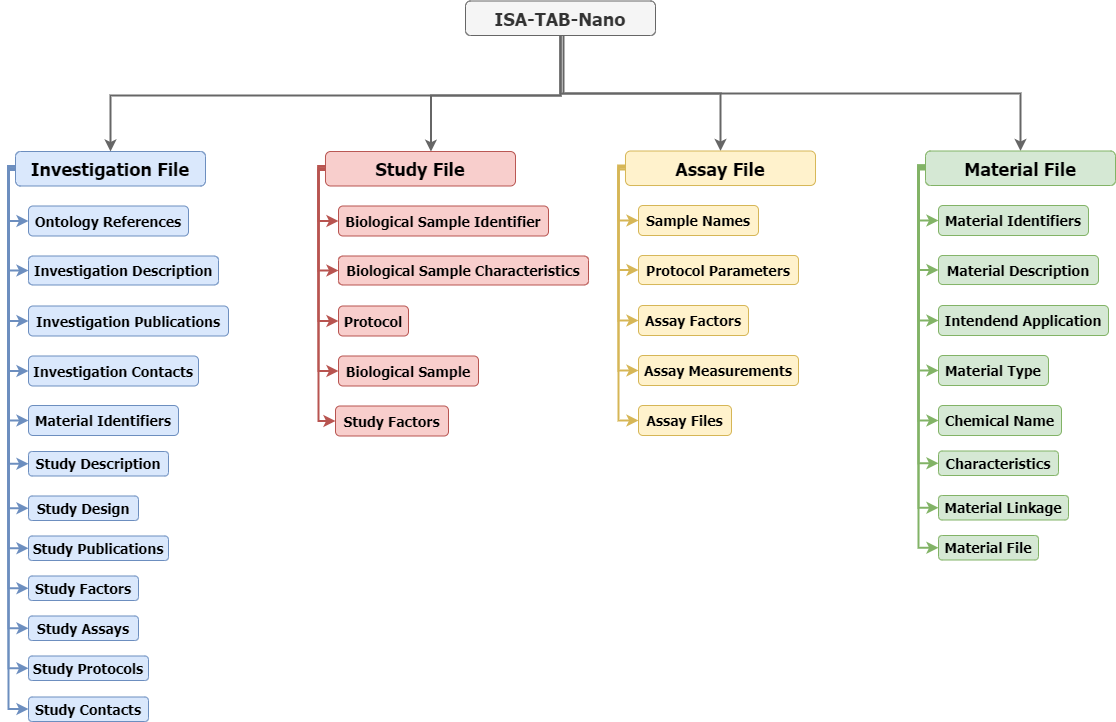


**Figure S2: Representation of the ISA-TAB Nano specification structure** ISA-TAB-Nano consists of four spreadsheet-based files including corresponding information.

**References**

1. Hastings J, Jeliazkova N, Owen G, Tsiliki G, Munteanu CR, Steinbeck C, et al. eNanoMapper: harnessing ontologies to enable data integration for nanomaterial risk assessment. J Biomed Semant 2015 61 [Internet]. BioMed Central; 2015 [cited 2021 Oct 16];6:1–15. Available from: https://jbiomedsem.biomedcentral.com/articles/10.1186/s13326-015-0005-5

2. Smith B, Ashburner M, Rosse C, Bard J, Bug W, Ceusters W, et al. The OBO Foundry: coordinated evolution of ontologies to support biomedical data integration. Nat Biotechnol 2007 2511 [Internet]. Nature Publishing Group; 2007 [cited 2021 Jul 27];25:1251–5. Available from: https://www.nature.com/articles/nbt1346

3. Uber Anatomy Ontology - Summary | NCBO BioPortal [Internet]. [cited 2021 Jul 27]. Available from: https://bioportal.bioontology.org/ontologies/UBERON

4. Mungall CJ, Torniai C, Gkoutos G V, Lewis SE, Haendel MA. Uberon, an integrative multi-species anatomy ontology. Genome Biol 2012 131 [Internet]. BioMed Central; 2012 [cited 2021 Jul 27];13:1–20. Available from: https://genomebiology.biomedcentral.com/articles/10.1186/gb-2012-13-1-r5

5. NanoParticle Ontology - Summary | NCBO BioPortal [Internet]. [cited 2021 Jul 27]. Available from: https://bioportal.bioontology.org/ontologies/NPO

6. Thomas DG, Pappu R V., Baker NA. NanoParticle Ontology for cancer nanotechnology research. J Biomed Inform. Academic Press; 2011;44:59–74.

7. Chemical Entities of Biological Interest Ontology - Summary | NCBO BioPortal [Internet]. [cited 2021 Jul 27]. Available from: https://bioportal.bioontology.org/ontologies/CHEBI

8. J H, G O, A D, M E, N K, V M, et al. ChEBI in 2016: Improved services and an expanding collection of metabolites. Nucleic Acids Res [Internet]. Nucleic Acids Res; 2016 [cited 2021 Jul 27];44:D1214–9. Available from: https://pubmed.ncbi.nlm.nih.gov/26467479/

9. BioAssay Ontology - Summary | NCBO BioPortal [Internet]. [cited 2021 Jul 27]. Available from: https://bioportal.bioontology.org/ontologies/BAO

10. Abeyruwan S, Vempati UD, Küçük-McGinty H, Visser U, Koleti A, Mir A, et al. Evolving BioAssay Ontology (BAO): modularization, integration and applications. J Biomed Semantics [Internet]. BioMed Central; 2014 [cited 2021 Jul 27];5:S5. Available from: /pmc/articles/PMC4108877/

11. Information Artifact Ontology - Summary | NCBO BioPortal [Internet]. [cited 2021 Jul 27]. Available from: https://bioportal.bioontology.org/ontologies/IAO

12. The IAO Community: The Information Artifact Ontology [Internet]. 2010 [cited 2021 Jul 27]. Available from: https://github.com/information-artifact-ontology/IAO/

13. Burgoon L. The AOPOntology: A Semantic Artificial Intelligence Tool for Predictive Toxicology. bioRxiv. 2018;276832.

14. Environment Ontology - Summary | NCBO BioPortal [Internet]. [cited 2021 Jul 27]. Available from: https://bioportal.bioontology.org/ontologies/ENVO

15. Buttigieg PL, Pafilis E, Lewis SE, Schildhauer MP, Walls RL, Mungall CJ. The environment ontology in 2016: bridging domains with increased scope, semantic density, and interoperation. J Biomed Semant 2016 71 [Internet]. BioMed Central; 2016 [cited 2021 Jul 27];7:1–12. Available from: https://jbiomedsem.biomedcentral.com/articles/10.1186/s13326-016-0097-6

16. Ontology for Biomedical Investigations - Summary | NCBO BioPortal [Internet]. [cited 2021 Jul 27]. Available from: https://bioportal.bioontology.org/ontologies/OBI

17. Bandrowski A, Brinkman R, Brochhausen M, Brush MH, Bug B, Chibucos MC, et al. The Ontology for Biomedical Investigations. PLoS One [Internet]. Public Library of Science; 2016 [cited 2021 Jul 27];11:e0154556. Available from: https://journals.plos.org/plosone/article?id=10.1371/journal.pone.0154556

18. Ontology of Adverse Events - Summary | NCBO BioPortal [Internet]. [cited 2021 Jul 27]. Available from: https://bioportal.bioontology.org/ontologies/OAE

19. He Y, Sarntivijai S, Lin Y, Xiang Z, Guo A, Zhang S, et al. OAE: The Ontology of Adverse Events. J Biomed Semant 2014 51 [Internet]. BioMed Central; 2014 [cited 2021 Jul 27];5:1–13. Available from: https://jbiomedsem.biomedcentral.com/articles/10.1186/2041-1480-5-29

20. Chemical Information Ontology - Summary | NCBO BioPortal [Internet]. [cited 2021 Jul 27]. Available from: https://bioportal.bioontology.org/ontologies/CHEMINF

21. Hastings J, Chepelev L, Willighagen E, Adams N, Steinbeck C, Dumontier M. The Chemical Information Ontology: Provenance and Disambiguation for Chemical Data on the Biological Semantic Web. PLoS One [Internet]. Public Library of Science; 2011 [cited 2021 Jul 27];6:e25513. Available from: https://journals.plos.org/plosone/article?id=10.1371/journal.pone.0025513

22. Phenotypic Quality Ontology - Summary | NCBO BioPortal [Internet]. [cited 2021 Jul 27]. Available from: https://bioportal.bioontology.org/ontologies/PATO

23. ISA-Tab format — ISA Model and Serialization Specifications 1.0 documentation [Internet]. [cited 2021 Jul 27]. Available from: https://isa-specs.readthedocs.io/en/latest/isatab.html

24. P R-S, M B, E M, N S, C T, K B, et al. ISA software suite: supporting standards-compliant experimental annotation and enabling curation at the community level. Bioinformatics [Internet]. Bioinformatics; 2010 [cited 2021 Jul 27];26:2354–6. Available from: https://pubmed.ncbi.nlm.nih.gov/20679334/

25. ISA-TAB-Nano - ICR Workspace - NCI Wiki [Internet]. [cited 2021 Jul 27]. Available from: https://wiki.nci.nih.gov/display/ICR/ISA-TAB-Nano#ISA-TAB-Nano-title

26. Thomas DG, Gaheen S, Harper SL, Fritts M, Klaessig F, Hahn-Dantona E, et al. ISA-TAB-Nano: A Specification for Sharing Nanomaterial Research Data in Spreadsheet-based Format. BMC Biotechnol 2013 131 [Internet]. BioMed Central; 2013 [cited 2021 Jul 27];13:1–15. Available from: https://bmcbiotechnol.biomedcentral.com/articles/10.1186/1472-6750-13-2

**
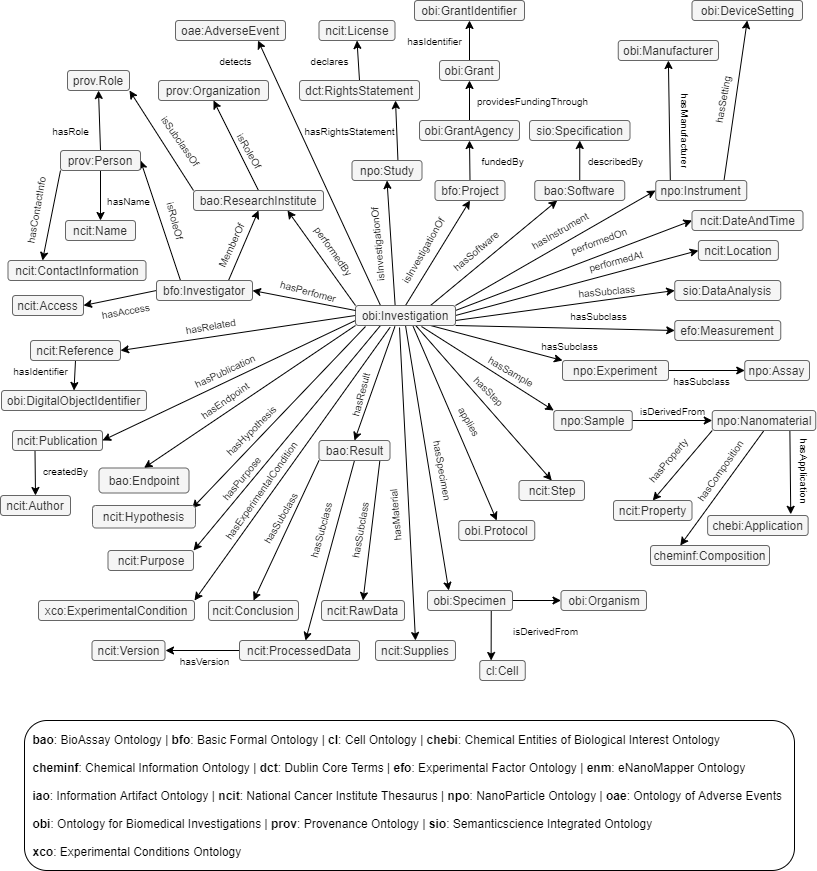
**

**Figure S3: Overview conceptualization an ontology based on the MIT** In accordance with the content focus of the MIT, entities that represent the investigations for the safety assessment of ENMs (*e.g.,* ENM characterization, *in vitro* and *in vivo* toxicology testing) the associated framework conditions and generated data will be defined by the ontology. A core entity would therefore be a class “Investigation” which summarizes this type of investigations (specified by subclasses „Physicochemical Characterization“, „Assay“, „In Vitro“ and „In Vivo“) that are carried out in the frame of a study (class “Study”) or funded project (class “Project”). Related funding details are then represented by the classes “Grant Agency”, “Grant” and “Grant Identifier”. Associated context and provenance information is captured by classes such as “Purpose”, “Hypothesis”, “Person”, “Research Institute“, “Date and Time” and “Location”. Details that characterise the involved scientists are modeled by the classes “Contact information”, “Name” and „Role“ (*e.g.,* specified by subclasses such as „Investigator“, “Assay Provider”, etc.). Reference information relevant to the planned study design or for publication purposes and digital resources such as a resulting publication or a method description are described by classes such as “Reference”, “Publication” and “Protocol”. The classes “Author” and “Digital Object Identifier'' are provided for the associated descriptive information. Related administrative details such as rights, license information and access authorization are covered by the classes "Rights Statement", "License" and "Access". Subject-specific information on experiment conditions, execution and evaluation, including the associated endpoints (*e.g.,* physicochemical or toxicological), examined sample material, used model organism and instruments as well as the resulting findings such as identified adverse events and generated raw and derived data are described by classes such as "Procedure", "Experimental Condition'', "Sample", "Specimen", "Instrument", "Endpoint" (specified by subclasses such as “Physchem Property Endpoint” and “Toxicological Endpoint”), „Data Analysis“, "Result", “Adverse Event” and "Raw Data". The classes mentioned here can be mapped with corresponding counterparts from existing ontologies.

**Table S2: Challenges for new approach methodologies (NAMs)** Parameters for lung, intestine and skin, and corresponding important model-specific parameters for nanosafety testing.

| **Lung^1^** | **Intestine^2^** | **Skin^3^** |
| --- | --- | --- |
| **Biological model information** | | |
| **Cell or tissue model** | | |
| e.g., mono/co-cultures of A549, monocyte-derived dendritic cells (MDDCs), monocyte-derived macrophages (MDMs) | e.g., mono/co-cultures of Caco-2 cells, THP-1 cells, HT29-MTX-E12 cells, Raji B cells | (3D) -human skin models e.g. EpiDermTM , EpiKuitis™ |
| **Justification of the biological model** | | |
| e.g., exhibiting properties representing alveolar epithelial type II cells and macrophages | e.g., exhibiting properties representing colon cells producing mucus and mimicking healthy and inflamed intestine | e.g., a model of reconstructed human epidermis with normal human-derived keratinocytes, multiple viable cell layers and a functional stratum corneum |
| **Molecular features** | | |
| mono/co-culture info (e.g., cell density, transwell), differentiation status, culture conditions apical and basal, surfactant/mucus production | mono/co-culture info (e.g., cell ratios, density, transwell), proliferation/differentiation status, culture conditions apical and basal, mucus production, stable vs. inflamed co-culture | culture conditions transwell, cell viability value as quality criteria |
| **Exposure information** | | |
| **Dose metrics** | | |
| e.g., aerosol concentration, (agglomerate) size distribution, exposure duration, deposited mass dose (quartz-balance), deposited number dose (TEM/SEM), internal dose/uptake (microscopical methods; ICP-MS, Effective Particle Density, and the Relevant In Vitro Dose (RID), etc.) | e.g., size/agglomeration behaviour, stability in experimental medium (e.g., DLS analysis), dissolution rate in test model, *in vitro* sedimentation modelling (e.g., ISDD: In vitro sedimentation, diffusion, and dosimetry model), internal dose/uptake | e.g., investigation and justification of the administered dose, vehicle, delivered dose, *in vitro* sedimentation, diffusion, dosimetry, agglomeration, transdermal adsorption |
| **Application of the ENM** | | |
| apical as aerosol like liquid drops or powder | apical as dispersion or emulsion to simulate dietary conditions | apical as dispersion or powder |
| **Investigation of model specific requirements** | | |
| aerosol type, aerosol generation, aerosol humidity, aerosol dilution, aerosol volume, ALI-chamber conditions (temperature, relative humidity, flowrate, electrostatic precipitation) | ingestion simulator including parameter description related to *in vivo* conditions to simulate digestive processes of the mouth, stomach and intestine regarding proteins and salts, pH value, temperature, and incubation time | reference positive control to induce skin corrosion or skin irritation, UV radiation |

| **Behaviour of the ENMs inside the system** | | |
| --- | --- | --- |
| e.g., uniformity, agglomeration, mass concentration and mass deposition on grids for transmission electron microscopy (TEM) placed on the transwell membranes or onto the cells, measured with a quartz crystal microbalance as a real-time indicator for the deposition of droplets | e.g., uniformity, agglomeration, mass concentration and mass deposition for transmission electron microscopy (TEM) onto the cells, DLS including information on medium temperature, viscosity, density, and height | e.g., investigation for skin penetration using transmission electron microscope (TEM) |
| **Endpoints (quality control)** | | |
| e.g., membrane integrity (e.g., TEER), ENM deposition and uptake | e.g., membrane integrity, ENM uptake, cytokine secretion, mucus production | e.g., ENM uptake, skin related toxicity that includes irritation, sensitization, corrosion, histopathological analyses and phototoxicity |

**^1^** Herzog et al. 2014, Diabate et al. 2021; **^2^** DeLoid et al 2017, Kämpfer et al. 2021; **^3^** Kim et al. 2016, Tang et al. 2018

**Table S3: Glossary of terms used in the present article**

| **Term** | **Definition** |
| --- | --- |
| **Controlled vocabulary** | A standardized and organized arrangement of words and phrases presented as alphabetical lists of terms or as thesauri and taxonomies with a hierarchical structure of broader and narrower terms [1].  An example of a controlled vocabulary is Medical Subject Headings (MESH) from the National Library of Medicine used to describe biomedical and health-related information [2]. |
| **Curation boundaries** | A curation boundary forms the interface between data stores e.g., between stores of the private, shared, and public domain. At this boundary, data and metadata to be transferred are specified, access controls or persistent identifiers are assigned [3]. |
| **Database** | A logical collection of information that is interrelated and that is managed and stored as a unit, for example in the same computer file [4].  In the context of this work, databases containing published data on physicochemical or in vitro/in vivo toxicity studies are of particular interest. |
| **Description standard** | A set of rules and guidelines that define the type and structure of meta(data) information used to represent resources (e.g., research data from scientific experiments). |
| **Labeled field** | Used in the context of this work as a synonym for both the MIT or ISA-TAB-Nano metadata elements and the eNanoMapper ontology classes.  A distinction is made between labeled fields, which only provide guidance for grouping of the subordinate labeled fields and have no content and content-bearing labeled fields, which are to be filled with content and to be made searchable for later reuse. |
| **Mapping** | In the context of this work, mapping is the process of performing a one-to-one assignment from elements of a source schema (e.g., MIT of this work) to elements of a target schema (e.g., eNanoMapper ontology or ISA-TAB nano standard) in order to identify equivalents and to determine interoperability between the schemas. |
| **Metadata** | Structured information that helps to identify, describe, manage, and grant access to resources of all kinds (e.g., audio or video files, books, images, etc.) by standardized schemas. Metadata can be of different types [5, 6].  In the context of this work, both descriptive metadata (e.g., title, author or abstract to describe scientific literature in general, such as publications) and subject-specific metadata (e.g., sample, endpoint or method to describe the nanosafety experiments conducted and the associated research data such as physicochemical data or in vitro/in vivo toxicity data) are of particular interest. |
| **Metadata schema** | Specification that defines metadata input fields and their properties, called elements and attributes, and the rules for using the elements to describe resources in machine-readable form. Metadata schemas can differ in the type and number of metadata elements and in the encoding (e.g., JSON, RDF/XML or XML format) [7].  Examples for metadata schemas are Dublin Core, developed and maintained by the Dublin Core Metadata Initiative (DCMI) [8], or DataCite from the DataCite International Consortium [9].  In the context of this work, metadata schemas are of interest as they enable standardised acquisition of nanosafety-based metadata across the entire course of research. |
| **Modules** | Main components of the minimal information table (MIT). Every module containing descriptive information and quality criteria related to the defined module name and therefore forms a closed information unit. Further information’s can be added as module subdivisions according to the modular principle. |
| **Module subdivisions** | Subjects to modules and containing complementary information. |
| **Ontology** | Formal representation of a knowledge domain, consisting of a vocabulary and rules for its composition, expressed in a machine-readable and -interpretable format such as OWL, RDF/XML or Turtle. It defines hierarchically ordered concepts that represent generic terms describing objects of the abstract or the real world (also called entities, e.g., person, place, or subject) based on common properties (= terminological knowledge). They are modelled in the form of classes (e.g., class "material"), which can be further specified by inserting subclasses (e.g., subclass “ENM”). By assigning instances or elements, (e.g., “titanium dioxide”) these can be provided with concrete values (= assertional knowledge level). Properties then establish the relationship between these classes (object properties, e.g., “nanomaterial isSubclassOf material” or “titanium dioxide isInstanceOf nanomaterial”) or provide further specifications (annotation properties, e.g., “hasIdentifier” or datatype properties, e.g., “hasParticlesize 100 nm”, which assign numbers or string values). [10] Each class and property consists of a label (e.g. “nanoparticle”) and a Uniform Resource Identifier (URI; e.g., “http://purl.bioontology.org/ontology/npo#NPO_707“) for its unique identification.  Examples of ontologies are Basic Formal Ontology (BFO), developed as part of the project Forms of Life funded by the Volkswagen Foundation [11] or the Ontology for biomedical Investigations (OBI), maintained by the OBI consortium [12]. |
| **Knowledge graph** | A knowledge graph is built on an ontology and can be considered as a network that provides interlinked factual and comprehensive knowledge on a specific topic. To make this type of knowledge machine-readable and -interpretable, the entities of an underlying ontology are organized by nodes that are connected via relationships. To distinguish them, they are labeled and provided with a URI, as is the case with the classes and properties of an ontology [13].  Examples for knowledge graphs are Wikidata [13,14], which models the data made available in Wikipedia, Wikimedia Commons, and other wikis of the Wikimedia movement or Google's knowledge graph [13], which is used to model and relate structured information on the web, for example about people, places, events, products, etc., to enrich search results within the Google search engine or to link various data sources. |
| **Uniform Resource Identifier (URI)** | A compact sequence of characters that identifies an abstract or physical resource. A URI can be further classified as a locator (URL), a name, or both. The term "Uniform Resource Locator" (URL) refers to the subset of URIs that, in addition to identifying a resource, provide a means of locating the resource by describing its primary access mechanism (e.g., its network "location"). [15] |

**References**

[1] https://op.europa.eu/de/web/eu-vocabularies/controlled-vocabularies

[2] <https://www.nlm.nih.gov/mesh/meshhome.html>

[3] <https://www.ands.org.au/guides/curation-continuum>

[4] Handbook on Geographic Information Systems and Digital Mapping, Studies in Methods, Series F, No. 79, United Nations Department of Economic and Social Affairs, Statistics Division, New York, 2000, Annex VI - Glossary.

[5] Riley, J. (2017). Understanding metadata. *Washington DC, United States: National Information Standards Organization (http://www. niso. org/publications/press/UnderstandingMetadata. pdf)*, *23*.

[6] Greenberg J. Understanding metadata and metadata schemes. Cat Classif Q [Internet]. Taylor & Francis Group; 2005 [cited 2021 Jul 27];40:17–36. Available from: https://www.tandfonline.com/doi/abs/10.1300/J104v40n03_02

[7] https://data.europa.eu/sites/default/files/d2.1.2_training_module_1.4_introduction_to_metadata_management_en_edp.pdf

[8] https://www.dublincore.org/schemas/xmls/

[9] https://schema.datacite.org/

[10] Noy NF, Mcguinness DL. Ontology Development 101: A Guide to Creating Your First Ontology. [cited 2021 Jul 27]; Available from: [www.unspsc.org](http://www.unspsc.org)

[11] http://basic-formal-ontology.org/

[12] The Ontology for Biomedical Investigations, PLoS One. 2016 Apr 29;11(4):e0154556. doi: 10.1371/journal.pone.0154556. eCollection 2016.

[13] Andreas, B., & Helmut, N. (2020). The Knowledge Graph Cookbook Recipes That Work. Edition mono/monochrom, Vienna.

[14] https://www.wikidata.org/wiki/Wikidata:Main_Page

[15] T. Berners-Lee; R. Fielding; L. Masinter. [Uniform Resource Identifier (URI): Generic Syntax](http://www.ietf.org/rfc/rfc3986.txt). January 2005. RFC. URL:<http://www.ietf.org/rfc/rfc3986.txt>
